# Insights Into Tribal‐Level Adaptive Evolution and Phylogeny in Soricinae From Mitogenome of the Chinese Endemic *Sorex cansulus*

**DOI:** 10.1002/ece3.73766

**Published:** 2026-06-09

**Authors:** Tao Wen, Xiujia Wu, Qian Lu, Wenyu Song, Wenge Dong

**Affiliations:** ^1^ Key Laboratory of Ectoparasite Systematics and Evolution of Yunnan Provincial Education Department, Institute of Pathogens and Vectors Dali University Dali China

**Keywords:** evolutionary rate, mitogenome, phylogeny, *Sorex cansulus*, Soricinae

## Abstract

*Sorex cansulus* is a Near Threatened shrew species endemic to China. To elucidate the adaptive evolution and evolutionary relationships of five tribes of the subfamily Soricinae, this study presented the morphology and complete mitogenome of *S. cansulus* . Codon usage bias analyses indicated that natural selection was the predominant force shaping its mitochondrial protein‐coding genes, with the exception of the *atp8* gene. We performed a comparative mitogenome analysis of 43 species across 13 genera and 5 tribes. The evolutionary rates (*K*_a_/*K*_s_) of 13 protein‐coding genes across all tribes were significantly less than 1, implying strong purifying selection and evolutionary conservation. Notably, tribes with more similar *K*_a_/*K*_s_ ratios exhibited closer evolutionary relationships. Phylogenetic trees yielded identical topologies and strongly supported the monophyly of Soricinae (PP = 1.00; BS = 100%). Both divergence time estimation and phylogenetic trees supported key taxonomic revisions: the reclassification of *Episoriculus fumidus* to the genus *Pseudosoriculus*; the elevation of *Blarinella griselda* to the genus *Parablarinella*, and the recognition of *S. cansulus* and *Sorex sinalis* as distinct sister species. However, some internal nodes within tribes showed relatively low nodal support, possibly due to incomplete taxon sampling or rapid radiative evolution. All five tribes retained the typical mammalian mitogenome organization, with the notable exception of *Sorex daphaenodon* , *Sorex tundrensis* , and *Sorex araneus* , which possessed an extra *trnW* gene (23 tRNA genes in total). These three species shared this unique feature and formed a distinct well‐supported clade, the implications of which for their mitochondrial function and adaptation warrant further research.

## Graphical abstract

This study reveals that natural selection is the main force shaping the mitochondrial protein‐coding genes of zokors, with all five populations exhibiting strong purifying selection. Phylogenetic analysis and divergence time estimation strongly support the monophyly of the subfamily Soricinae, leading to key taxonomic revisions, including the reclassification of several species. The study also identified a unique lineage composed of three species of the genus Sorex, characterized by the presence of an additional trnW gene.

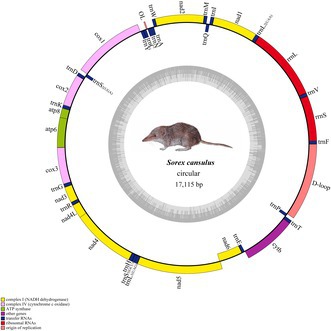

## 1 Introduction

Soricidae, comprising 448 species across 26 genera, is the most species‐rich family within the order Eulipotyphla (Bover et al. 2018; Wang 2019). It is currently divided into three subfamilies: Soricinae, Crocidurinae, and Myosoricinae (Wilson and Mittermeier 2018). Living shrew species are widely distributed across Europe, Asia, Africa, and the Americas, occupying a wide variety of habitats and lifestyles, including terrestrial, semi‐aquatic, semi‐fossorial, scansorial, and psammophilic (Hutterer 1985). Shrews prey upon a wide diversity of invertebrates, thereby playing a crucial role in maintaining food web stability and ecological balance (Churchfield et al. 2004; Van de Perre et al. 2019). Beyond their ecological importance, shrews also serve as ideal models for biogeographic studies (Quérouil et al. 2003). Furthermore, shrews harbor an exceptionally diverse virome, including many viruses typically associated with invertebrates. The documented multiorgan distribution of these viruses within shrews suggests that they may act as important intermediate hosts, facilitating the cross‐species transmission of viruses from invertebrates to vertebrates (Chen et al. 2023).

The subfamily Soricinae, commonly known as red‐toothed shrews, derives its name from the colored iron pigments present in its tooth enamel (Bover et al. 2018). This feature is present in most species within the subfamily, with the exception of white‐toothed shrews such as *Chimarrogale styani* , *Chimarrogale leander* and *Nectogale elegans* . Members of the subfamily are important zoonotic reservoirs for a variety of emerging viruses (Arai et al. 2012; Chen et al. 2022; Kang et al. 2010; Song et al. 2009). The subfamily Soricinae comprises 6 tribes, 13 genera, and 181 species, exhibiting high morphological diversity and distinct anatomical adaptations. *S. cansulus* (Thomas, 1912), a monotypic species with no recognized subspecies (Liu 2023), belongs to the subfamily Soricinae, and is endemic to China (Jiang 2024; Wei et al. 2022). This species is a relatively small in body size. Its dorsal fur is grayish brown and unstriped, while its ventral fur is light grayish brown. The tail is dark brown dorsally and light yellowish brown ventrally, with a clear boundary. For many years, *S. cansulus* was documented only from its type locality and adjacent areas in southern Gansu Province. Subsequent records have substantially expanded its known range. Smith and Xie (2009) collected *S. cansulus* in regions of southern Gansu near Qinghai and eastern Tibet, although precise location details were not provided. Song et al. (2021) collected the species in the Baima Snow Mountain and Langdu Mountain areas of northwestern Yunnan Province. Huang et al. (2022), when reviewing samples collected between 2011 and 2018, found that *S. cansulus* was identified in samples captured in Sichuan, Shaanxi, and Qinghai provinces. Pavlova et al. (2021) collected *S. cansulus* in Songpan and Zoige, Sichuan Province. *S. cansulus* was assessed as Data Deficient (DD) on *the IUCN Red List* in 2015 owing to its extreme elusiveness and the difficulty of field sampling (https://www.iucnredlist.org). However, it was listed as Near Threatened (NT) on *the Chinese Red List* in 2020 due to its extremely narrow distribution, small population size, and ongoing habitat degradation caused by logging and cultivation (Jiang 2021).

The mitogenome serves as a classical marker widely used in studies of adaptive evolution, phylogenetics, and population genetics (Avise et al. 1987; Ballard and Whitlock 2003), providing crucial evidence for unraveling the population history, lineage divergence, and speciation events of shrews. However, mitogenome data and relevant literature remain relatively scarce for the subfamily Soricinae in China (Wang 2019), primarily owing to their small body size and highly elusive nature, which make them difficult to capture. The paucity of mitogenome data has hindered the exploration of phylogenetic relationships and taxonomic status within the subfamily Soricinae at the mitogenome level, resulting in a poor understanding of their evolutionary history. Such knowledge gaps present significant challenges for reconstructing macroevolutionary patterns (He et al. 2021; Willows‐Munro and Matthee 2011). In this study, we described the morphological characteristics of *S. cansulus* and sequenced its complete mitogenome. By integrating these new data with mitogenome sequences from 43 species across 5 tribes and 13 genera of the subfamily Soricinae retrieved from the NCBI database, we investigated the adaptive evolution and phylogenetic relationships among different tribes. Our research aims to establish a more robust evolutionary framework to enhance our understanding of the origin and diversification of the subfamily Soricinae.

## 2 Materials and Method

### 2.1 Specimen Collection and Identification

**FIGURE 1:**
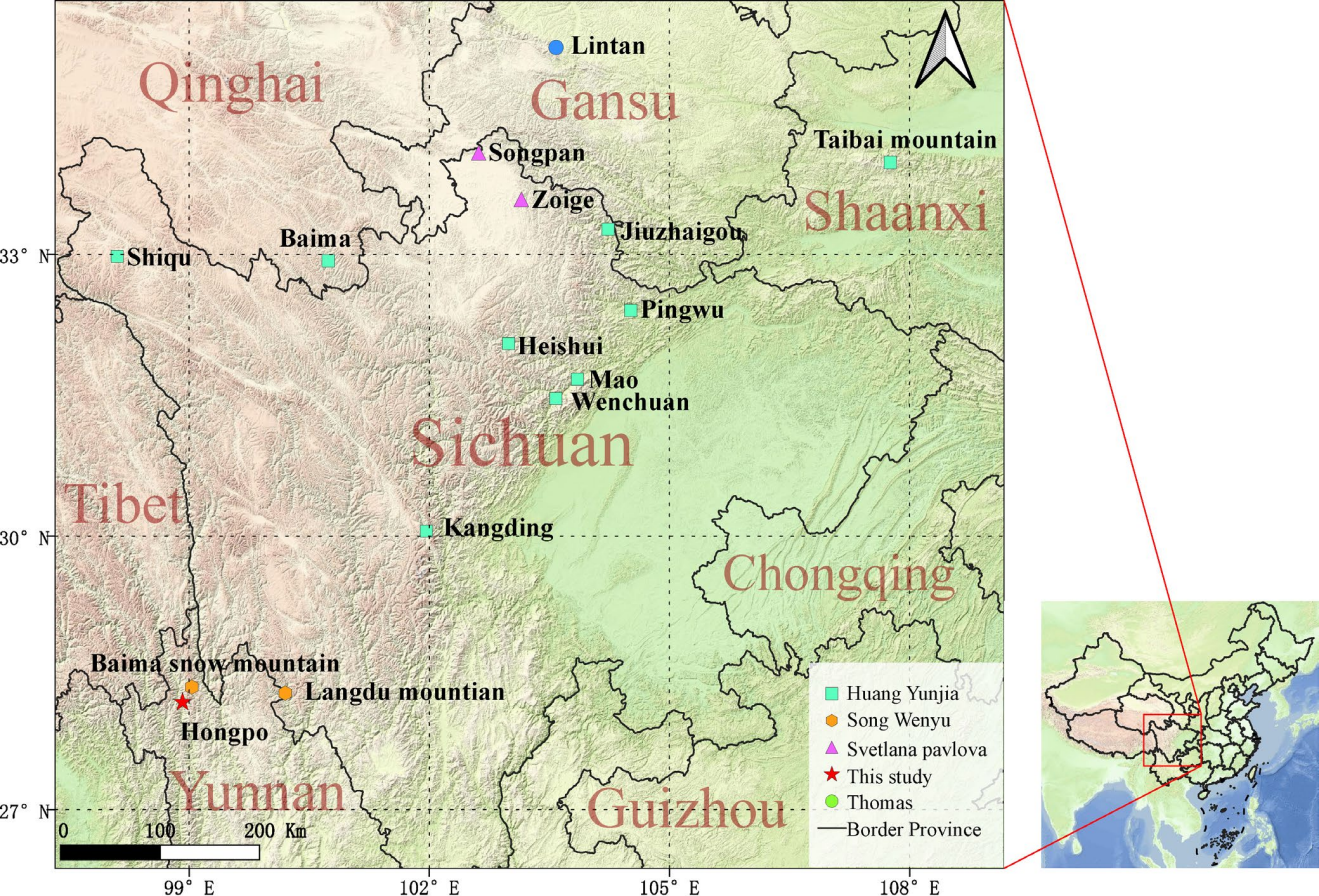
Distribution map of *Sorex cansulus* collection sites. Legend: Locality reported by Huang et al. (2022) (green square); locality reported by Song et al. (2021) (orange polygon); type locality Thomas (Wilson and Mittermeier 2018) (blue circle); locality reported by Pavlova et al. (2021) (purple triangle); and collection site of this study (red star). The visualization of the figure was performed using QGIS v3.40.14 and Adobe Illustrator v29.4.

In 2024, one adult female individual of *S. cansulus* was collected in Hongpo Village, Deqin County, Diqing Tibetan Autonomous Prefecture, Yunnan Province, China (28.31° N, 98.94° E) at an altitude of 4361 m. The specimen was captured using Sherman traps baited with unsweetened rolled oats (Figure 1).

Species identification was performed by integrating morphological and molecular approaches. The following morphological measurements were recorded in the field: body weight (g), body length (mm), tail length (mm), hindfoot length (mm), and ear length (mm). Morphological assessment was primarily based on characteristics such as body size, pelage color, cranial morphology, and dental formula. Molecular confirmation was achieved by sequencing the mitochondrial *cytb* gene using the following primers (L14725‐hsw1: 5′‐ATGACATGAAAAATCATCGTTGT‐3′ and H15915‐hsw1: 5′‐TCYCCATTTCTGGTTTACAAGACC‐3′). All species identifications were consistent with the taxonomic descriptions in *the Catalog of mammals in China* (Wei et al. 2025) and *the Handbook of the Mammals of the World* (Wilson and Mittermeier 2018). Muscle, liver, and spleen tissues were dissected and preserved in 95% ethanol in Eppendorf tubes, and stored at −80°C for subsequent analysis. Voucher specimens were deposited at the Institute of Pathogens and Vectors, Dali University (Dali, China), with the voucher number HP2405388. All experimental procedures involving *S. cansulus* were reviewed and approved by the Laboratory Animal Ethics Committee of Dali University.

### 2.2 DNA Extraction, Mitogenome Sequencing and Analysis

Genomic DNA was extracted from muscle tissue using the DNeasy Blood & Tissue Kit (QIAGEN). A paired‐end library (350 bp insert) was prepared and sequenced on an Illumina NovaSeq 6000 platform. Raw reads were filtered with fastp v1.0 (Chen 2025) to remove reads with > 5% ambiguous bases or > 50% bases with Q ≤ 5, and adapters were trimmed. Clean reads were assembled de novo using SPAdes v3.14.1 with k‐mers: 21, 45, 65, 85, 105. The complete mitogenome was extracted from the assembly graph using Bandage (Wick et al. 2015) and validated by read mapping with BWA (Li 2013) and coverage depth calculation using SAMtools (Li et al. 2009) (Figure S1). Assembly accuracy was further verified with Geneious Prime v11.1.5 (Kearse et al. 2012). Annotation was performed with MitoZ v2.3 (Meng et al. 2019); the annotated mitogenome was deposited in GenBank (accession number: PX208754). Nucleotide composition, codon usage bias, and strand asymmetry (AT‐skew, GC‐skew) were calculated using CodonW v1.4.2 (Peden 1999). Using RStudio v4.3.1, we analyzed nucleotide diversity (Pi), Correspondence analysis (COA), parity rule 2 (PR2), effective number of codons plot (ENC), neutrality plot, and relative synonymous codon usage (RSCU) for the 13 protein‐coding genes (PCGs). K2P genetic distances for *cytb* were computed using MEGA12 (Kumar et al. 2024). The circular map of the mitogenome was drawn with OGDraw (Greiner et al. 2019). Finally, evolutionary rates (*K*_a_/*K*_s_) for the 13 PCGs were calculated using KaKs‐Calculator 3.0 (Zhang 2022) based on the newly sequenced mitogenome and 43 shrew mitogenomes from 13 genera (Table S1), with *Parascaptor leucura* as outgroup.

### 2.3 Phylogenetic Analysis

We reconstructed the phylogeny of the subfamily Soricinae using the newly sequenced mitogenome of *S. cansulus* and 43 additional species from 5 tribes and 13 genera that are available in GenBank (Sayers et al. 2025). *P. leucura* and *Scaptonyx fusicauda* were selected as outgroups. A concatenated matrix of the 13 PCGs and 2 rRNA genes (PCGRNA dataset) was assembled. Multiple sequence alignment was performed with MAFFT v7.313 (Katoh et al. 2002) and optimized with MACSE v2.06 (Ranwez et al. 2018) to preserve reading frames. For alignment filtering, Gblocks v0.19b (Talavera and Castresana 2007) with default parameters was used to process the PCG alignment to remove poorly aligned positions and gaps. For the rRNA alignment, trimAl v1.2 (Capella‐Gutiérrez et al. 2009) was employed with default settings. Substitution saturation was assessed with DAMBE v7.3.32 (Xia 2018); the Iss value was significantly lower than Iss.c, confirming the dataset's suitability for phylogenetic inference. Phylogenetic trees were reconstructed using both Maximum Likelihood (ML) and Bayesian Inference (BI) in PhyloSuite v1.2.3 (Xiang et al. 2023; Zhang et al. 2020). The best‐fit model (GTR + I + G) was selected by ModelFinder v2.2.0 (Kalyaanamoorthy et al. 2017). ML analysis was performed with IQ‐TREE v2.2.2.7 (Nguyen et al. 2015) using 1000 ultrafast bootstrap replicates; nodal support was assessed as bootstrap (BS) values. BI analysis was conducted with MrBayes v3.2.6 (Ronquist and Huelsenbeck 2003) under the same model, with two independent runs, each with four MCMC chains for 200,000 generations, sampling every 1000 generations, and discarding the first 25% as burn‐in. Convergence was verified (ESS > 200). Nodal support was assessed as posterior probability (PP). Trees were visualized with ChiPlot (Xie et al. 2023) and edited in Adobe Illustrator 2025 v29.4.

### 2.4 Divergence Time Estimation

Divergence times were estimated using PhyloSuite v2 with the MCMCtree plugin (Zhao et al. 2025). The analysis was based on the phylogenetic tree and the concatenated alignment. Parameters were configured via MDGUI, the graphical interface of MCMCTree. A correlated rates model (relaxed molecular clock) and the approximate likelihood method (FAST mode) were applied. The root age was constrained to a minimum of 61.10 Ma (RootAge > 61.10). Additional fossil calibration points were set according to the TimeTree database and relevant literature (Table S2). Priors were set as following: rgene_gamma = 2.00–20.00, kappa_gamma = 6.00–2.00; substitution model = AUTO (allowing MCMCTree to select the best‐fitting model). All other parameters remained at their default values. The MCMC chain was run for 200,000 generations, sampling every 10 generations, with the first 25% discarded as burn‐in. Convergence was assessed using MCMCTracer with ESS > 200 for all parameters. Divergence times were annotated onto the maximum clade credibility (MCC) tree using TreeAnno, and the time tree was visualized with ChiPlot and edited in Adobe Illustrator 2025 v29.4.

## 3 Result

### 3.1 Morphological Characteristics of *S. cansulus*

**FIGURE 2:**
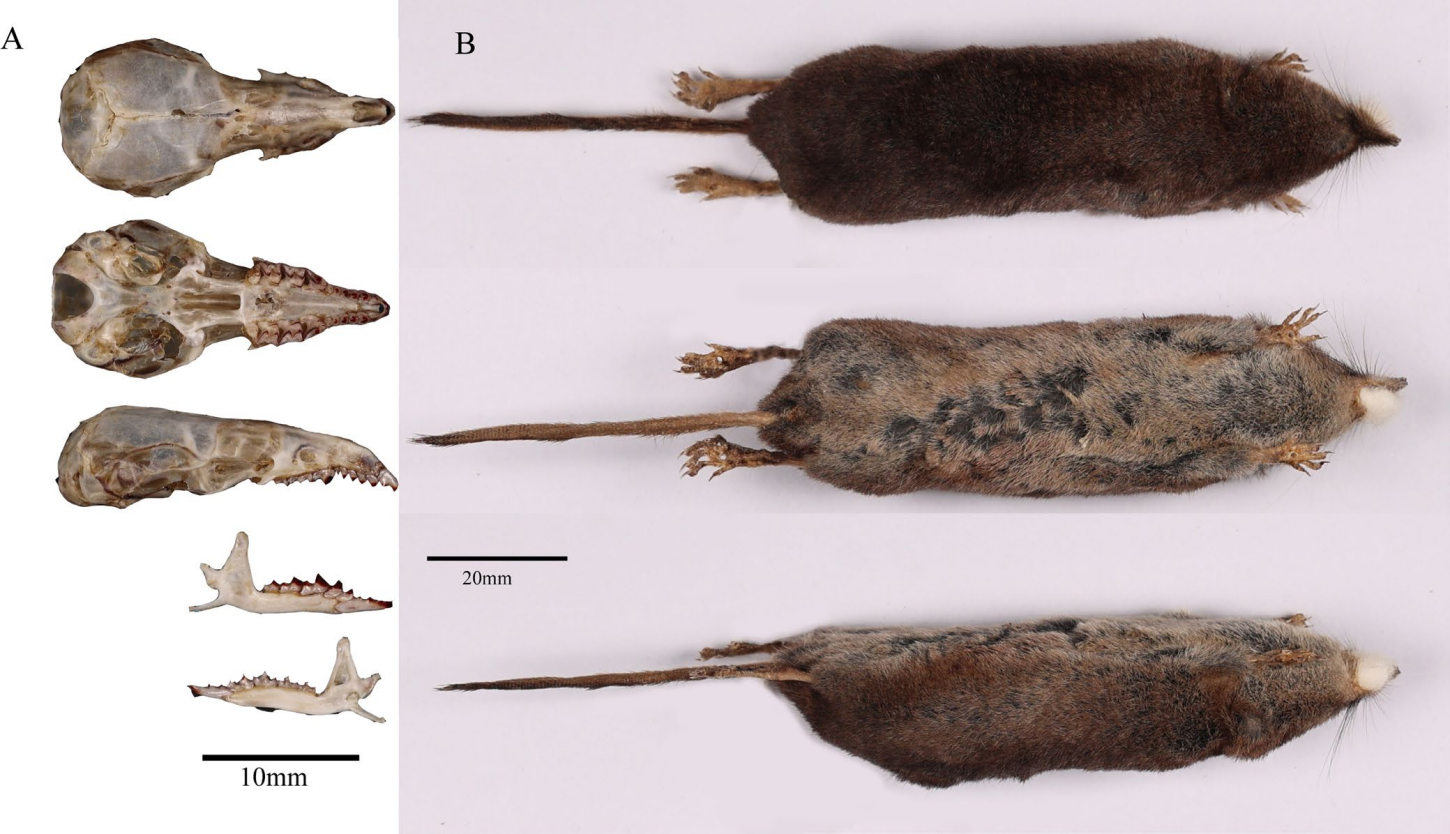
Morphological characteristics of *Sorex cansulus* (HP2405388). (A) Dorsal, ventral, and lateral views of the cranium, and bilateral views of the mandible; (B) Dorsal, ventral, and lateral views of the specimen.

The specimen was collected from Hongpo Village, Deqin County, Diqing Tibetan Autonomous Prefecture, Yunnan Province, China. Species description: An adult female specimen of *S. cansulus* (ID: HP2405388) was measured as follows: body weight 9.2 g, head‐body length 79 mm, tail length 52 mm, hindfoot length 14 mm, and ear length 4 mm. Its dorsal fur is grayish brown without stripes; its ventral fur is light grayish brown; dorsal surfaces of all four feet are brownish white. The tail is sharply bicolored, dark brown dorsally and light yellowish brown ventrally, and its length accounts for 65.8% of the head‐body length (Figure 2B). Moreover, this specimen is larger in body size than that reported for *S. cansulus* by Wilson and Mittermeier (2018).

### 3.2 Cranial and Dental Morphology

The skull is relatively high and not flattened; premaxilla, maxilla, and nasal bones are slender; the braincase is nearly rounded. Dental formula: 1.5.1.3/1.1.1.3 = 32. There are five upper unicuspid teeth. The occlusal surface of unicuspid teeth is wider than long. The second and third unicuspid teeth are roughly equal in size, but slightly smaller than the first unicuspid tooth; the fourth unicuspid tooth is slightly smaller than the third; the fifth unicuspid tooth is vestigial. The mandible is somewhat robust. Pigmentation is restricted to the cusps of the teeth, which are a deep chestnut‐red. The cutting edge of the lower incisor has two inconspicuous depressions (Figure 2A).

### 3.3 Mitogenome Structure of *S. cansulus*

**FIGURE 3:**
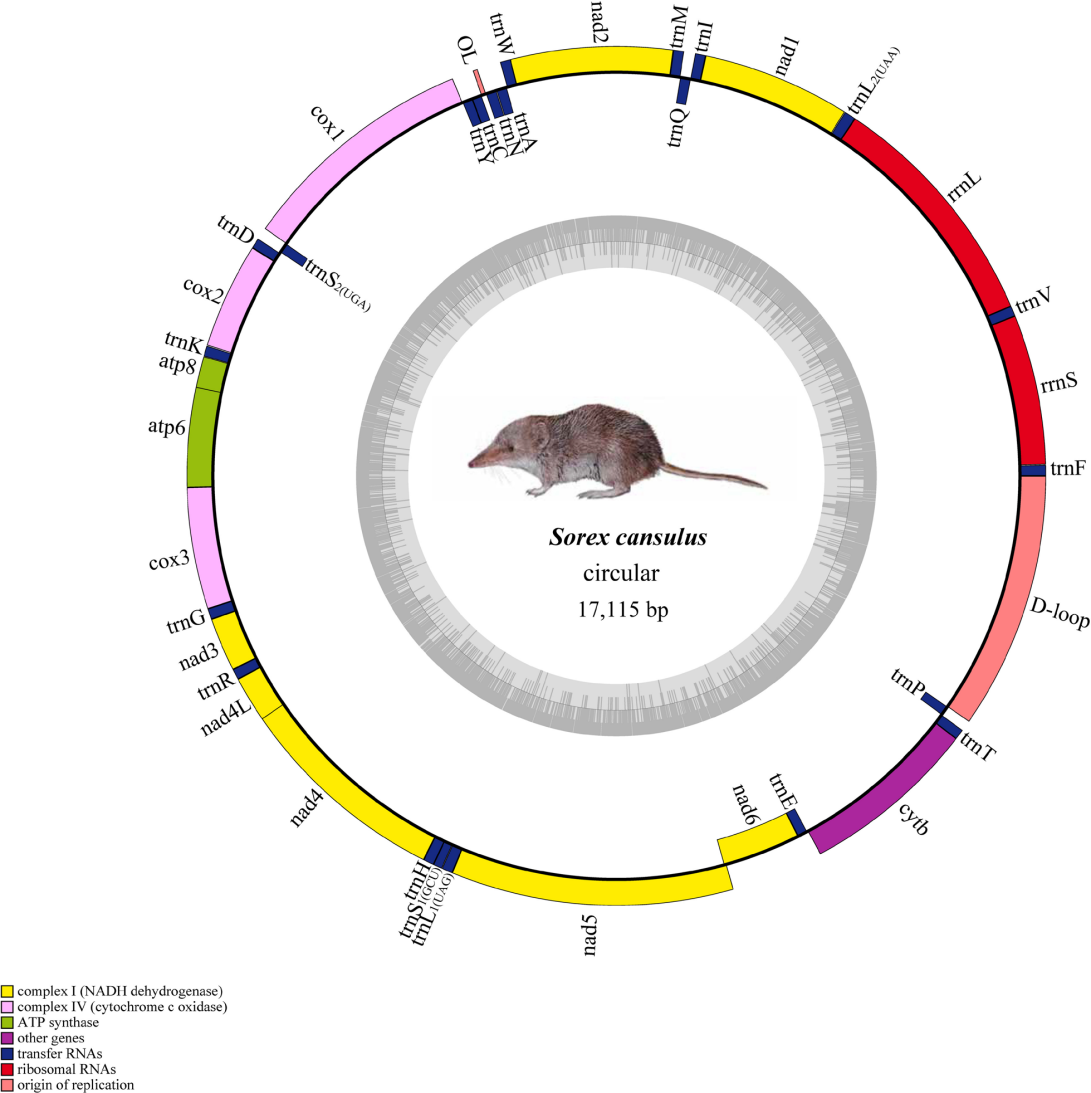
The circular map of the *Sorex cansulus* mitogenome. The morphological illustration of *Sorex cansulus* (inset) is adapted from Jiang (2024, 156).

The mitogenome of *S. cansulus* is 17,115 bp in length and exhibits the organization of the typical mammalian mitogenome. It encodes 37 genes: 13 PCGs, 22 tRNA genes, and 2 rRNA genes, as well as L‐strand replication origin (OL) and a D‐loop region (Figure 3). Three major tRNA gene clusters were identified: *trnI*‐*trnQ*‐*trnM*, *trnW*‐*trnA*‐*trnN*‐*trnC*‐*trnY*, and *trnH*‐*trnS*_*1(GCU)*_‐*trnL*_*1(UAG)*_. The OL (33 bp) is embedded within the W‐A‐N‐C‐Y tRNA gene cluster (between *trnN* and *trnC*), forming a stem‐loop structure that is essential for L‐strand replication. Gene distribution on the strands follows the typical mammalian pattern: the L‐strand encodes nine genes (*trnQ*, *trnA*, *trnN*, *trnC*, *trnY*, *trnS*_*2(UGA)*_, *trnE*, *trnP*, and *nad6*), while the remaining 28 genes are located on the H‐strand.

The mitogenome of *S. cansulus* contains seven overlapping regions, ranging from 1 to 43 bp in length. The longest overlap (43 bp) occurs between *atp8* and *atp6*, which is a typical feature of mammalian mitogenomes. Additionally, there are 10 intergenic spacers, ranging from 1 to 6 bp. The largest spacer (6 bp) is located between *trnS*_*2(UGA)*_ and *trnD* (Table 1).

**TABLE 1:** Organization of the *Sorex cansulus* mitogenome.

Gene	Strand	Position (bp)	Size (bp)	Intergenic nucleotides (bp)	Codon start/stop
*trnF*	H	1–69	69	0	
*rrnS*	H	72–1039	968	2	
*trnV*	H	1040–1106	67	0	
*rrnL*	H	1107–2674	1568	0	
*trnL*_*2(UAA)*_	H	2675–2749	75	0	
*nad1*	H	2752–3707	956	2	ATG/TA‐
*trnI*	H	3708–3776	69	0	
*trnQ*	L	3774–3846	73	−3	
*trnM*	H	3847–3915	69	0	
*nad2*	H	3916–4959	1044	0	ATA/TAG
*trnW*	H	4958–5025	68	−2	
*trnA*	L	5030–5099	70	4	
*trnN*	L	5102–5174	73	2	
OL	H	5175–5207	33	0	
*trnC*	L	5208–5274	67	0	
*trnY*	L	5275–5342	68	0	
*cox1*	H	5344–6888	1545	1	ATG/TAA
*trnS*_*2(UGA*_ *)*	L	6889–6958	70	0	
*trnD*	H	6965–7032	68	6	
*cox2*	H	7033–7716	684	0	ATG/TAA
*trnK*	H	7720–7788	69	3	
*atp8*	H	7790–7993	204	1	ATG/TAA
*atp6*	H	7951–8631	681	−43	ATG/TAA
*cox3*	H	8631–9414	784	−1	ATG/T‐‐
*trnG*	H	9415–9484	70	0	
*nad3*	H	9485–9831	347	0	ATC/TA‐
*trnR*	H	9832–9900	69	0	
*nad4L*	H	9901–10,197	297	0	ATG/TAA
*nad4*	H	10,191–11,568	1378	−7	ATG/T‐‐
*trnH*	H	11,569–11,637	69	0	
*trnS*_*1(GCU)*_	H	11,638–11,696	59	0	
*trnL*_*1(UAG)*_	H	11,698–11,767	70	1	
*nad5*	H	11,768–13,588	1821	0	ATA/TAA
*nad6*	L	13,572–14,108	537	−17	ATG/TAA
*trnE*	L	14,109–14,177	69	0	
*cytb*	H	14,183–15,322	1140	5	ATG/AGG
*trnT*	H	15,322–15,389	68	−1	
*trnP*	H	15,390–15,456	67	0	
D‐loop	H	15,457–17,115	1659		

*Note:* Positive values indicate intergenic spacers; negative values indicate overlaps.

The *Sorex cansulus* mitogenome shows a high AT content (62.3% AT, 37.7% GC). Its strand asymmetry is reflected by an AT skew value of 0.057 and a GC skew value of −0.301 (Table S3). The D‐loop region (1659 bp), located between *trnP* and *trnF*, contains conserved motifs essential for replication initiation and acts as the primary regulatory site for mitochondrial replication and transcription. The tRNA genes are interspersed among protein‐coding and rRNA genes. The ribosomal RNA genes *rrnS* and *rrnL* are located between *trnF* and *trnL*_*2(UAA)*_ and are separated by the *trnV* gene.

### 3.4 Structural Features of tRNAs and rRNAs in the *S. cansulus* Mitogenome

**FIGURE 4:**
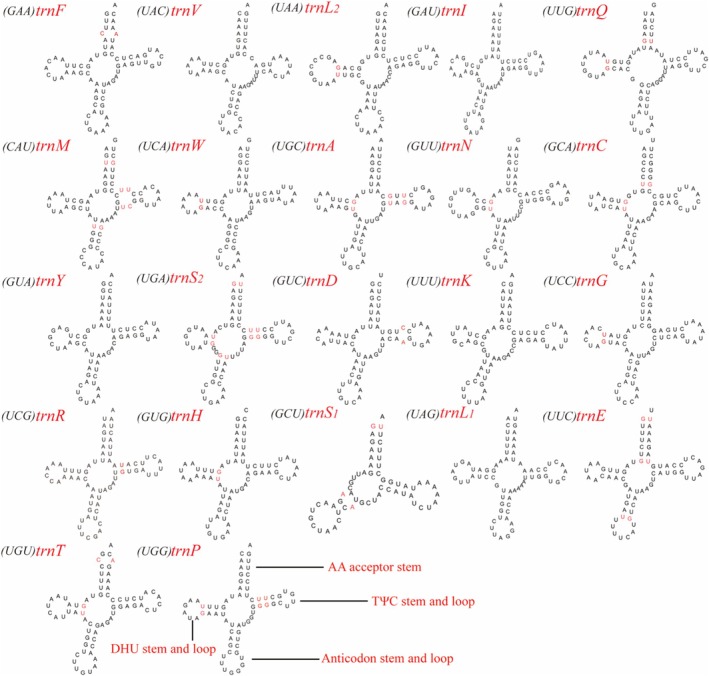
Predicted cloverleaf secondary structures of 22 tRNA genes in the *Sorex cansulus* mitogenome (GenBank accession No. PX208754). Anticodons are indicated in parentheses and non‐Watson–Crick pairings are highlighted in red. tRNA structures were predicted using tRNAscan‐SE v2.0 and ARWEN v1.2, and secondary structures were visualized with Adobe Illustrator 2025 v29.4.

The *S. cansulus* mitogenome encodes 22 tRNA genes (8 on the L‐strand, 14 on the H‐strand) and 2 rRNA genes (*rrnS*: 968 bp; *rrnL*: 1568 bp) on the H‐strand. The lengths of the tRNA genes range from 59 bp (*trnS*_*1(GCU)*_) to 75 bp (*trnL*_*2(UAA)*_) (mean ± standard deviation: 68.9 ± 2.99 bp; Table 1). All tRNA genes fold into the typical cloverleaf secondary structure except for *trnS*_*1(GCU)*_, which lacks the dihydrouridine (DHU) arm (Figure 4). Secondary structure predictions identified 34 non‐Watson‐Crick base pairings across 17 tRNA genes (Figure 4).

The majority of these non‐canonical pairings are G‐U wobble pairs (28 occurrences across 15 tRNA genes). The remaining six mismatches comprise A‐C, U–U, A‐A, A‐G and C‐U pairs. These atypical pairings were distributed across the tRNA arms as follows: the acceptor arm (8 mismatches), the DHU arm (11 mismatches), the TΨC arm (6 mismatches), and the anticodon arm (4 mismatches). In contrast, *trnV*, *trnI*, *trnY*, *trnK*, and *trnS*_*2(UAG)*_ exhibit exclusively canonical Watson‐Crick base pairing. The conserved lengths of the DHU stem (2–4 bp) and the TΨC stems are mostly 5 bp, except for *trnQ*, *trnC*, and *trnS*_*2(UAG)*_ (4 bp) and *trnV* (3 bp), reflecting constraints essential for translational accuracy (Figure 4).

### 3.5 Protein‐Coding Genes, Codon Usage, and Genetic Diversity of *S. cansulus*

**FIGURE 5:**
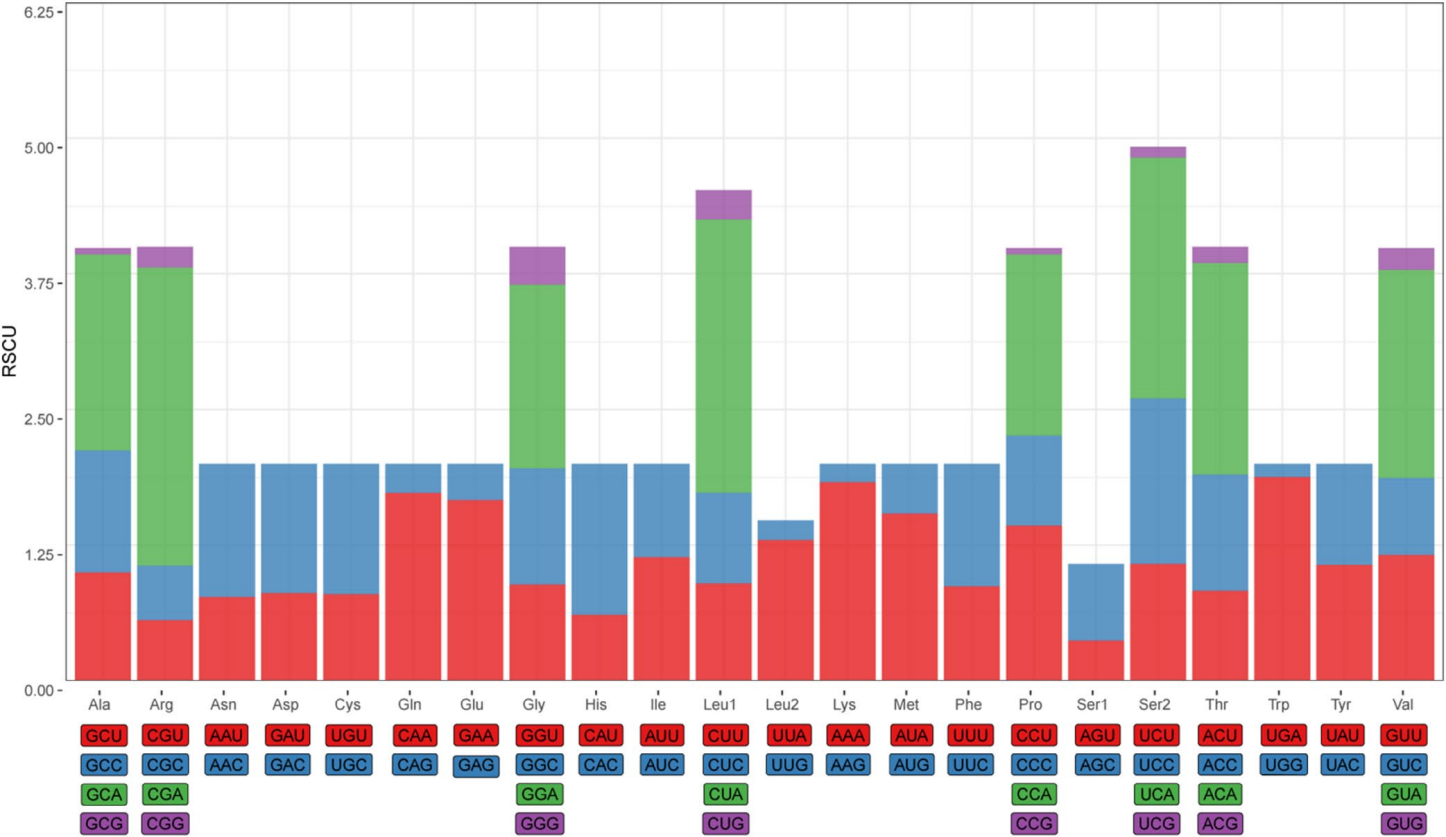
Relative synonymous codon usage (RSCU) of the 13 PCGs in the *Sorex cansulus* mitogenome.

The length of the 13 PCGs in the *S. cansulus* mitogenome is 11,418 bp, accounting for 66.7% of the total mitogenome (17,115 bp). The nucleotide composition of PCGs is A = 32.9%, T = 29.4%, C = 24.5%, G = 13.2%, with a high AT content of 62.3%, a positive AT skew (AT skew = 0.057), and a negative GC skew (GC skew = −0.301) (Table S3). All 13 PCGs use the typical ATN start codon, whereas four types of stop codons are identified: incomplete stop codons T‐‐ (*cox3*, *nad4*) and TA‐ (*nad1*, *nad3*); TAG (*nad2*); AGG (*cytb*); and the canonical TAA (the remaining seven PCGs) (Table 1). Analysis of RSCU across 3795 codons reveals a strong bias (Figure 5 and Table S4). Twenty‐nine high‐usage codons (RSCU > 1) account for 71.8% (2726/3795) of the total. Among these, 12 are markedly overrepresented codons (RSCU > 1.6, occurring 1272 times in total), including CUA (Leu), CGA (Arg), ACA (Thr), and UCA (Ser). Conversely, 17 underrepresented codons (RSCU < 0.6, appearing 230 times in total) may reflect translational selection against energetically costly amino acids.

The pronounced codon usage bias observed in the *S. cansulus* mitogenome indicates strong natural selection acting on translation efficiency and accuracy (Plotkin and Kudla 2011; Quax et al. 2015). Furthermore, the evolution of the mitogenome is dominated by purifying selection, as evidenced by the *K*_a_/*K*_s_ ratios for all PCGs, which are significantly less than 1. These values range from a minimum of 0.006 in *cox1* to a maximum of 0.207 in *atp8* (Figure S2, Table 2).

**TABLE 2:** Selection pressure analysis of protein‐coding genes (PCGs) in the *Sorex cansulus* mitogenome, using *Parascaptor leucura* (NC_056332) as outgroup.

Gene	*K*_a_/*K*_s_	*K*_a_	*K*_s_
*cox1*	0.006	0.027	4.286
*cox3*	0.008	0.031	3.897
*cytb*	0.009	0.035	4.105
*nad1*	0.012	0.051	4.063
*cox2*	0.014	0.054	3.830
*atp6*	0.031	0.043	1.409
*nad6*	0.034	0.117	3.480
*nad4L*	0.043	0.079	1.827
*nad3*	0.045	0.097	2.156
*nad5*	0.051	0.129	2.514
*nad4*	0.081	0.122	1.514
*nad2*	0.092	0.204	2.209
*atp8*	0.207	0.133	0.642

**FIGURE 6:**
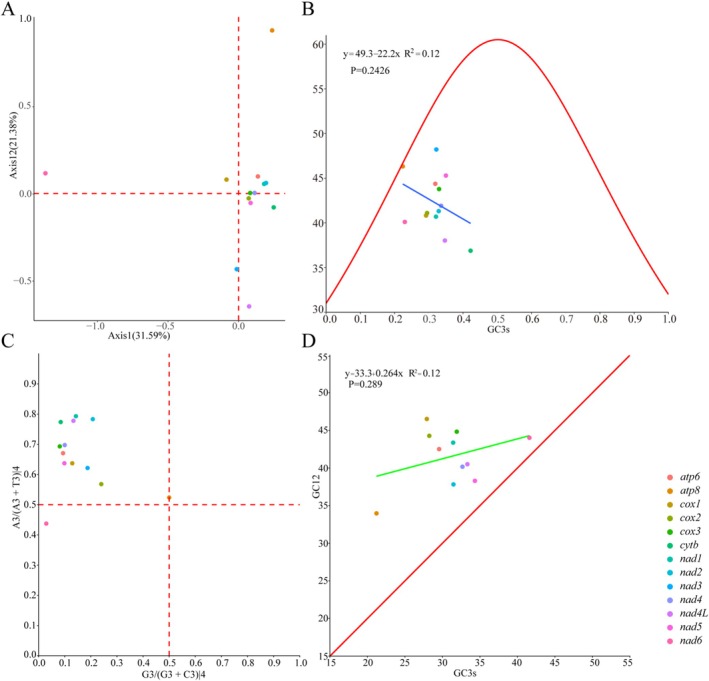
Multivariate analysis of codon usage bias for the 13 PCGs in the *Sorex cansulus* mitogenome. (A) Correspondence analysis (COA) based on codon frequency. Axis 1: The first principal component; Axis 2: The second principal component. (B) Effective number of codons (ENC) plot (GC3s: GC content at the third position of synonymous codons; Nc: The effective number of codons; the linear equation *y* = 49.3–22.2*x* is shown as a blue line in the figure, with *R* ^2^ indicating correlation and *p* indicating significant difference). (C) Parity Rule 2 (PR2) plot for evaluating purine/pyrimidine preference. (D) Neutrality curve. GC12: Average GC content at the first and second positions of synonymous codons. The linear equation *y* = 33.3 + 0.264*x* is shown as a green line.

Codon usage bias in the *S. cansulus* mitogenome was comprehensively analyzed using correspondence analysis COA, ENC, PR2, and neutral curve analysis (Figure 6). COA analysis showed that most PCGs were scattered on the right side of Axis 1, with Axis 1 accounting for 31.59% of the total variation and Axis 2 accounting for 38% (Figure 6A). The ENC values of these genes ranged from 36.95 (*cytb*) to 48.28 (*nad3*). With the exception of *atp8*, which was close to the expected standard curve, all other points fell below it (Figure 6B). This finding implies that *atp8* is more strongly influenced by mutation, whereas natural selection plays a dominant role in the mutation‐drift balance for the remaining genes. PR2 analysis revealed that 11 PCGs clustered in the upper‐left quadrant, indicating a strong preference for A/C bases. In contrast, *nad6* exhibited a T/C preference. Notably, *atp8* was positioned near the central point of the parity rule (Figure 6C). Neutral curve analysis indicated a regression slope of 0.264 for *S. cansulus* , corresponding to a relative neutrality rate of 26.4%. This indicates that mutation pressure accounts for 26.4% of codon usage bias, while selection pressure contributes the remaining 73.6% (Figure 6D). Since the regression slope for *S. cansulus* is less than 1, it can be concluded that this species is subject to stronger selection pressure.

### 3.6 Comparative Analysis of Evolutionary Rates (*K*_a_/*K*_s_) Among Five Tribes of the Subfamily Soricinae

**FIGURE 7:**
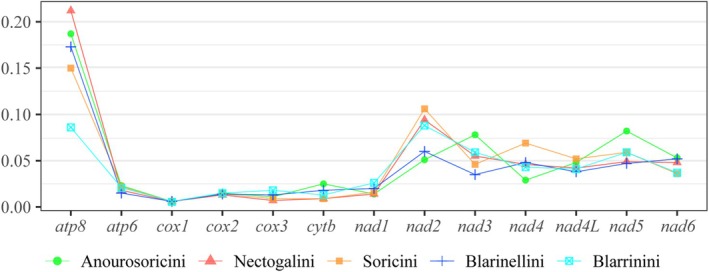
Comparison of *K*_a_/*K*_s_ ratios for the 13 PCGs across five tribes of the subfamily Soricinae.

The *K*_a_/*K*_s_ values for the 13 PCGs across five tribes of the subfamily Soricinae were all significantly less than 1 (Table S5; Figure 7). Among them, five genes (*atp8*, *nad2*, *nad5*, *nad3*, and *nad4*) showed slightly greater variation in *K*_a_/*K*_s_ values among tribes, although all values remained below 0.25. The greatest variation was observed in *atp8* (0.162). In contrast, the remaining eight genes exhibited minimal fluctuations in evolutionary rate (*K*_a_/*K*_s_) across tribes (all below 0.05), with *cox1* showing the least variation (0.006).

In Nectogalini, Anourosoricini, Soricini, and Blarinellini, the highest *K*_a_/*K*_s_ ratio was in *atp8* and the lowest in *cox1*. In Blarinini, the highest ratio was in *nad2* and the lowest in *cox1*. Across all five tribes, *atp8* exhibited the highest mean evolutionary rate and *cox1* the lowest, consistent with strong purifying selection on mitochondrial protein‐coding genes. The mean rates in descending order: *atp8* > *nad2* > *nad5* > *nad3* > *nad4* > *nad6* > *nad4L* > *atp6* > *nad1* > *cytb* > *cox2* > *cox3* > *cox1*.

The average evolutionary rate for the PCGs across different tribes of the subfamily Soricinae, ranked from highest to lowest, was as follows: Anourosoricini (0.048) > Nectogalini (0.047) > Soricini (0.046) > Blarinellini (0.041) > Blarinini (0.039) (Table S5).

### 3.7 Phylogenetic Analysis

**FIGURE 8:**
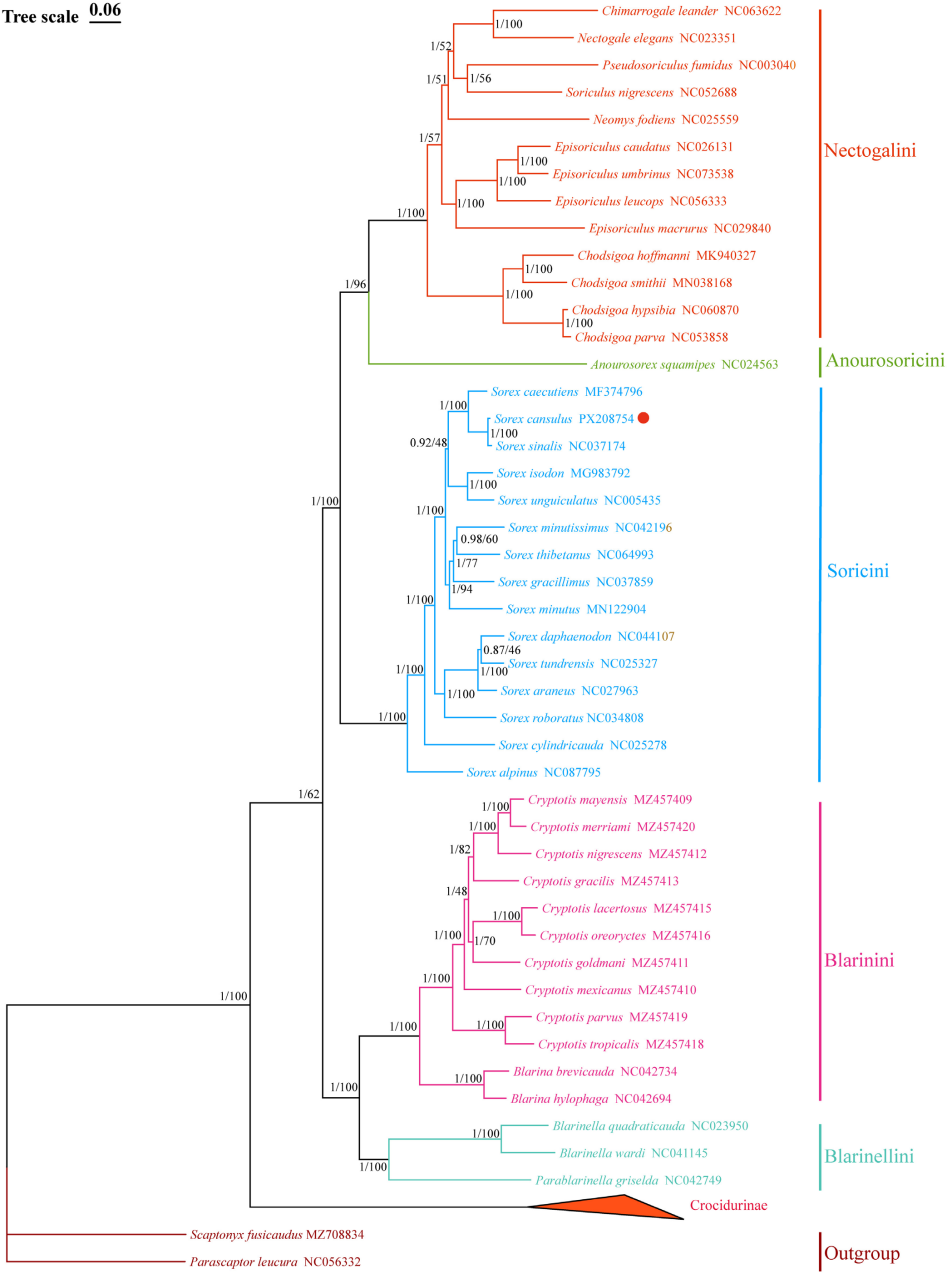
Maximum likelihood (Figure S3) and Bayesian inference trees of Soricinae inferred from the PCGRNA dataset. The target species of this study is marked with a red dot. Tribes are highlighted with distinct colors. *Parascaptor leucura* and *Scaptonyx fusicauda* were used as outgroups. Node labels show posterior probability (PP)/bootstrap support (BS) (%) values.

We assessed substitution saturation using DAMBE, which yielded Iss < Iss.c, indicating that the nucleotide sequences of PCGs are not saturated and are therefore suitable for phylogenetic reconstruction (Table S6). The maximum likelihood (ML) and Bayesian inference (BI) phylogenetic trees were constructed based on the concatenated PCGRNA dataset. Following sequence alignment, the length of PCGs was 11,372 bp, and the length of rRNAs was 2243 bp, with total concatenated length of 13,615 bp. The ML and BI trees shared identical topologies. In both trees, the subfamily Soricinae was recovered as a monophyletic group with strong nodal support (PP = 1.00, BS = 100%). The phylogenetic position of *S. cansulus* within the subfamily Soricinae also received high nodal support (PP = 1.00, BS = 100%; Figure 8). The relationships among genera within the subfamily Soricinae were resolved as follows: ((((((((*Pseudosoriculus* + *Soriculus*) + (*Chimarrogale* + *Nectogale*)) + *Neomys*) + *Episoriculus*) + *Chodsigo*a) + *Anourosorex*) + *Sorex*) + ((*Parablarinella* + *Blarinella*) + (*Blarina* + *Cryptotis*))). Within the subfamily Soricinae, the phylogenetic relationships among some tribes remain unresolved, as indicated by low support values at several nodes. The tribe Soricini contains solely the genus *Sorex*, which forms a sister group to the clade comprising the tribes Nectogalini and Anourosoricini. The genus *Sorex* was strongly supported as a monophyletic group, with high nodal support (PP = 1.00; BS = 100%).

Within this genus, the 15 species of *Sorex* formed six distinct clades: (1) *S. cansulus* + *S. sinalis* + *S. caecutiens* ; (2) *Sorex isodon* + *Sorex unguiculatus* ; (3) *Sorex thibetanus* + *Sorex minutissimus* + *Sorex gracillimus* + *Sorex minutus* ; (4) *Sorex daphaenodon* + *Sorex tundrensis* + *Sorex araneus* + *Sorex roboratus* ; (5) *Sorex cylindricauda* ; (6) *S. alpinus* . Notably, the three species forming the fourth clade ( *S. daphaenodon* , *S. tundrensis* , and *S. araneus* ) shared a distinctive mitogenome organization (the presence of 23 tRNA genes, including an extra *trnW*). Species with similar mitogenome organization were clustered together in the phylogenetic tree.

Phylogenetic analysis strongly supported *S. cansulus* and *S. sinalis* as sister species (PP = 1.00; BS = 100%), confirming their close affinity and upholding their status as distinct species. The tree topology validated the recognition of *Pseudosoriculus* as a separate genus from *Soriculus*, with the two genera recovered as sister groups. Our results also supported elevating *Blarinella griselda* to the genus *Parablarinella* (as *Parablarinella griselda*). Furthermore, the phylogeny supported the independent taxonomic status of *S. cansulus* and confirmed that *Pseudosoriculus* and *Soriculus* were sister genera.

### 3.8 Divergence Time Estimation

**FIGURE 9:**
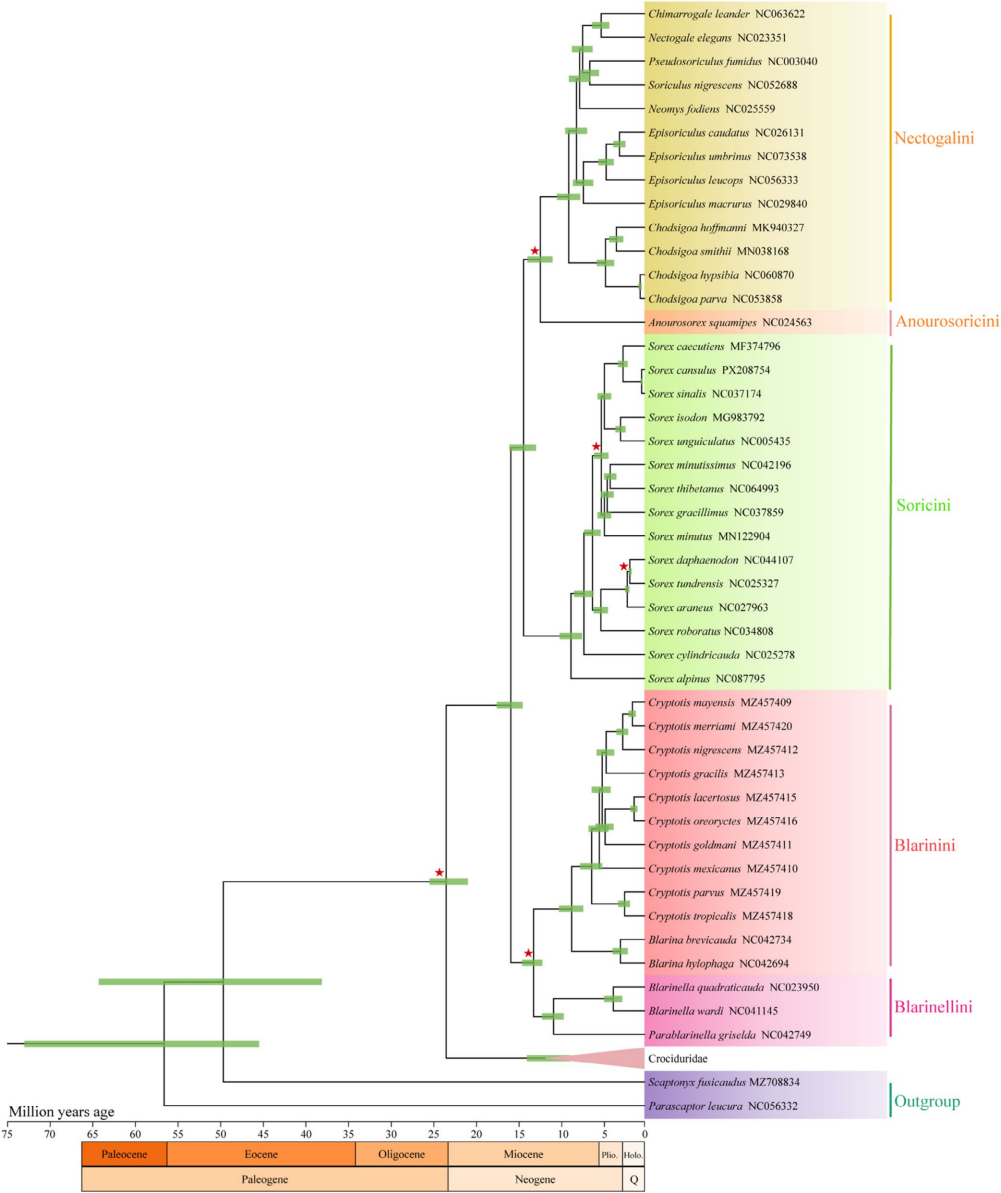
Time‐calibrated phylogenetic tree of the subfamily Soricinae (Eulipotyphla, Soricidae) inferred from mitogenomes using PhyloSuite v2. Nodes represent the median age of the most recent common ancestor (MRCA); bars at nodes indicate the 95% HPD intervals. Red pentagrams mark the positions of five fossil calibrations that were selected via multi‐step evaluation (Table S2).

Divergence time analysis revealed that the outgroup diverged earliest from the ingroup taxa at 49.41 Ma (95% HPD: 37.85–64.01 Ma; Figure 9). Among the sampled shrews, the first major divergence of primary lineages occurred at 23.26 Ma (95% HPD: 20.74–25.20 Ma), suggesting that the early divergence of extant major lineages began in the Early Miocene. Divergence within the subfamily Soricinae occurred at 15.67 Ma (95% HPD: 14.28–17.32 Ma), with several major clades separating at 11.98–14.35 Ma and 12.73–15.84 Ma, indicating that these lineages were largely established by the Middle Miocene. At higher taxonomic levels, crown‐group divergence times varied among genera. For example, the main divergence nodes of the *Sorex*‐related lineages were mostly concentrated around 8.62 Ma (95% HPD: 7.32–9.94 Ma). The divergence time among *Blarinella quadraticauda* , *Blarinella wardi* , and *Parablarinella griselda* was dated to approximately 10.67 Ma (95% HPD: 9.48–12.02 Ma), far exceeding typical interspecific divergence times. Similarly, the main crown‐group divergence time of the genus *Episoriculus* was estimated at 7.12 Ma (95% HPD: 6.01–8.90 Ma), whereas the common ancestor of *Episoriculus fumidus* and other congeners was dated to 7.99 Ma, also far exceeding average intrageneric divergence times (Figure 9).

## 4 Discussion

### 4.1 Mitogenome Structure and Evolutionary Conservation: From Single Species to Subfamily Level

This study is the first to report the complete mitogenome of *S. cansulus* , a Near Threatened species endemic to China, filling a significant gap in genomic evolutionary research for this taxon. Its mitogenome (17,115 bp) conforms to the typical mammalian pattern in terms of gene composition, arrangement, and strand distribution (Boore 1999), encoding a total of 37 genes (13 PCGs, 22 tRNA genes and 2 rRNA genes); of these, 9 genes are located on the L‐strand and 28 genes on the H‐strand. In addition, the OL region exhibits GC enrichment (51.5%)—a feature that may be attributed to reduced selective pressure for RNA primer binding during the initiation of replication on the L‐strand (Zardoya et al. 1995). The genome displays a high AT content (62.3%) and pronounced strand asymmetry (AT skew = 0.057, GC skew = −0.301), consistent with the pattern of strand‐specific deamination observed in other Soricidae species (Reyes et al. 1998).

Multivariate analyses (ENC plot, PR2 analysis and neutrality plot) revealed that the evolutionary trajectories of the PCGs in this species are not uniform. Although they are generally subject to strong purifying selection to maintain protein functional stability, they may also experience positive selection driving adaptive evolution (Shen et al. 2010). Genes encoding core subunits of the respiratory chain (e.g., *cox1‐3*, *cytb*) are under strong purifying selection constraints, whereas *atp8* exhibits a significantly relaxed constraint pattern. The higher evolutionary rate of *atp8* (*K*_a_/*K*_s_ = 0.207) may be attributed to its shorter gene length, weaker functional constraints, or a predominance of neutral amino acid substitutions (Ballard and Whitlock 2003; da Fonseca et al. 2008); in contrast, *cox1* shows the lowest rate (0.006), consistent with its crucial and irreplaceable function in the electron transport chain (Tsukihara et al. 1996). Notably, despite the prevalence of strong purifying selection in mammals (Bhardwaj et al. 2024), the overall evolutionary rate of the PCGs in *S. cansulus* is lower than the general mammalian level but comparable to that of other members of the subfamily Soricinae (Wang et al. 2025) (Table S7). This suggests that members of the subfamily are under exceptionally strong functional constraints at the mitochondrial level. In intertribal comparisons, the average *K*_a_/*K*_s_ value was highest in Anourosoricini (0.048) and lowest in Blarinini (0.039). However, the differences among tribes were generally small, and all *K*_a_/*K*_s_ values were considerably lower than 1, which warrants caution when interpreting these minor variations in average evolutionary rates across different tribes. Additionally, our data may imply a possible correlation: tribes with more similar average evolutionary rates tend to be more closely related in the phylogenetic tree. Overall, strong purifying selection appears to be the primary evolutionary mechanism maintaining the functional stability of mitochondrial genes in the subfamily Soricinae, which is consistent with the general principle that the more critical a function, the stronger the purifying selection (Cai et al. 2009; Shen et al. 2009).

tRNA genes exhibit extensive evolutionary conservation. In particular, *trnS*_*1(GCU)*_ lacks the DHU arm, a feature commonly observed in vertebrates. The 34 non‐Watson‐Crick base pairs, of which 28 are *G*‐*U* wobble pairs, may stabilize the secondary structure through low‐energy hydrogen bonds (Petersheim and Turner 1983) and could be modified into more stable canonical base‐pairing by RNA editing (Watanabe and Yokobori 2011). The three major tRNA gene clusters (*trnI*‐*trnQ*‐*trnM*, *trnW*‐*trnA*‐*trnN*‐*trnC*‐*trnY* and *trnH*‐*trnS*_*1(GCU)*_‐*trnL*_*1(UAG)*_) are highly conserved across vertebrates, further supporting the structural stability of the mitogenome in the subfamily Soricinae.

### 4.2 Phylogenetic Relationships and Taxonomic Revision: Temporal Framework of Tribal Divergence

The phylogenetic tree based on the PCGRNA matrix yielded highly congruent topologies from both BI and ML analyses. Our results robustly supported the monophyly of the subfamily Soricinae with high nodal support (PP = 1.00, BS = 100%). The tribe Soricini, represented solely by the genus *Sorex*, was reconstructed as the sister clade to the clade comprising Nectogalini and Anourosoricini. Several intra‐tribal nodes exhibited low support values, which may be attributed to insufficient taxon sampling or rapid radiative evolution, highlighting the need for further investigation to resolve these relationships.

Divergence time estimation revealed that diversification of major extant lineages within the subfamily Soricinae commenced in the Early Miocene (approximately 23.26 Ma; 95% HPD: 20.74–25.20 Ma), whereas tribal‐level lineages were largely established during the Middle Miocene (approximately 15.67 Ma; 95% HPD: 14.28–17.32 Ma). The crown‐group diversification of the tribe Soricini was dated to approximately 8.62 Ma (95% HPD: 7.32–9.94 Ma), a timeframe that coincides closely with episodes of climatic oscillation and habitat fragmentation across Eurasia in the Late Miocene.

This study provides key evidence supporting several taxonomic revisions: First, *Episoriculus fumidus* should be placed in the genus *Pseudosoriculus*. This species clusters with members of *Pseudosoriculus* in the phylogenetic tree rather than with those of *Episoriculus*, and its genetic distance is comparable to that of other species within *Pseudosoriculus* (Table S8). Furthermore, its divergence time (approximately 7.99 Ma) significantly exceeds the level of interspecific differentiation within the genus *Episoriculus*, further supporting its distinct generic status (Abramov et al. 2017; He et al. 2010). Second, *Blarinella griselda* should be elevated to the genus *Parablarinella*. Phylogenetically, this species is distinct from other species of the genus *Blarinella*, and its divergence time from *Blarinella* (about 10.67 Ma) far exceeds the typical interspecific differentiation level within the subfamily Soricinae. Additionally, the genetic distance between the two genera is significant (Table S9), which is consistent with the suggestions proposed by He et al. (2018) and Bannikova et al. (2019). Third, *S. cansulus* and *S. sinalis* cluster together as sister species. They form a well‐supported clade in the phylogenetic tree (PP = 1.00, BS = 100%), supporting their status as separate species.

### 4.3 Atypical Mitogenome Organization: Evolutionary Implications of *trnW* Gene Duplication

Within the tribe Soricini, the mitogenomes of three species, *S. daphaenodon* , *S. tundrensis* , and *S. araneus* , encode 23 tRNA genes, representing one additional *trnW* gene duplication compared to the typical mammalian mitogenome configuration. This rare structural feature corresponds to a stable clade in the phylogenetic tree, suggesting that it likely originated from a single *trnW* gene duplication event in the common ancestor of these three species.

The tRNA gene duplication is exceptionally rare in mammalian mitogenomes, and its functional significance remains unclear. Potential explanations include: (1) The redundant copy gradually degenerates or retains neutral variation under relaxed selection (Massey 2015); (2) The additional *trnW* gene may compensate for a higher demand for tryptophan in certain tissues or developmental stages (Hughes et al. 2023; Raval et al. 2023); (3) This structural feature may be associated with specific ecological adaptations of these three species, such as their distribution across Palaearctic high‐latitude regions (Song et al. 2025). The possibility that it represents a stochastic duplication event that was randomly retained cannot be ruled out; however, its stable presence across three species forming a monophyletic group suggests that it may have undergone some form of positive selection or functional differentiation. The molecular mechanisms and evolutionary drivers of this phenomenon warrant further investigation through expanded sampling and analysis at the nuclear genome level.

## 5 Conclusion

This study described the morphological characteristics of *S. cansulus* and sequenced its complete mitogenome. Comparative analyses were performed using data from 43 species across 13 genera and five tribes of Soricinae to elucidate adaptive evolution and phylogenetic relationships among these tribes. The mitogenome organization of *S. cansulus* was consistent with the typical mammalian pattern. Codon usage bias analysis (ENC plots, PR2 analysis, neutrality curve, and RSCU) indicated that natural selection was the primary driver shaping its codon usage patterns, with the exception of *atp8*. All five tribes exhibited typical mammalian mitogenome organization, except for *S. daphaenodon* , *S. tundrensis* , and *S. araneus* , which possessed an extra *trnW* gene (23 tRNA genes in total). These three species shared this unique feature and formed a distinct clade. Evolutionary rate (*K*_a_/*K*_s_) analysis revealed that all protein‐coding genes across five tribes were under strong purifying selection, reflecting evolutionary conservatism. Phylogenetic analysis strongly supported the monophyly of Soricinae. The diversification of the subfamily Soricinae dates back to 15.67 million years ago (95% HPD: 14.28–17.32 Ma). The divergence time and phylogenetic results of this study supported several key taxonomic revisions: reassigning *Episoriculus fumidus* to *Pseudosoriculus*; placing *Blarinella griselda* into a separate genus (*Parablarinella*); recognition of *S. cansulus* and *S. sinalis* as distinct sister species. However, phylogenetic relationships within some tribes remained incompletely resolved, likely due to insufficient taxon sampling or rapid radiative evolution. Collectively, these findings provide a more robust evolutionary framework for understanding the origin, adaptive evolution and diversification of the subfamily Soricinae.

## Author Contributions

**Tao Wen:** conceptualization (lead), methodology (lead), writing – original draft (lead), writing – review and editing (lead). **Xiujia Wu:** data curation (equal). **Qian Lu:** data curation (equal). **Wenyu Song:** conceptualization (equal), project administration (lead). **Wenge Dong:** project administration (lead), software (lead), writing – review and editing (lead).

## Funding

This work was supported by the National Natural Science Foundation of China (No. 32260152 to Wenge Dong; No. 32260277 to Wenyu Song) and the Research Foundation of the Department of Education of Yunnan Province (No. 2026Y1320 to Tao Wen).

## Ethics Statement

This study strictly adhered to the animal practice standards set by relevant national and/or local animal welfare agencies, and all animal capture agreements and procedures had been approved by the Animal Ethics Committee of Dali University (approval number: 2021‐P2‐162). All methods were carried out in accordance with relevant guidelines and regulations.

## Conflicts of Interest

The authors declare no conflicts of interest.

## Supporting information


**Figure S1:** Mitogenome sequencing depth and coverage map of *Sorex cansulus*.

**Figure S2:** Selection pressure analysis chart of 13 protein‐coding genes of *Sorex cansulus*.

**Figure S3:** Maximum Likelihood Phylogenetic Tree of the subfamily Soricinae.

**Table S1:** Species information.

**Table S2:** Fossil calibration points for divergence time trees.

**Table S3:** Nucleotide composition of the *Sorex cansulus* mitogenome.

**Table S4:** Codon frequency and relative rynonymous codon usage (RSCU) of protein‐coding genes (PCGs) in the *Sorex cansulus* mitogenome.

**Table S5:** Ka/Ks ratios of protein‐coding genes (PCGs) in 5 tribes of the subfamily Soricinae.

**Table S6:** Saturation test of nucleotide sequence for protein‐coding genes (PCGs) of *Sorex cansulus* .

**Table S7:** Ka/Ks comparison of protein‐coding genes (PCGs) among *Sorex cansulus* , *Dacnomys millardi* , and Soricinae.

**Table S8:** Genetic distance between *Pseudosoriculus* and *Episoriculus*.

**Table S9:** Genetic distance between *Parablarinella* and *Blarinella*.

## Data Availability

In this study, all datasets (Table S1) are available online. The database name and website are as follows: NCBI Database: https://www.ncbi.nlm.nih.gov/. For the complete mitogenome of *Sorex cansulus* (accession number: PX208754) https://www.ncbi.nlm.nih.gov/nucleotide/PX208754.1.
