# Supplementary figures and images for: Insights Into Tribal‐Level Adaptive Evolution and Phylogeny in Soricinae From Mitogenome of the Chinese Endemic Sorex cansulus
Source: Ecol Evol. 2026 Jun 9;16(6):e73766. doi: 10.1002/ece3.73766 (PMC13249582; doi:10.1002/ece3.73766)

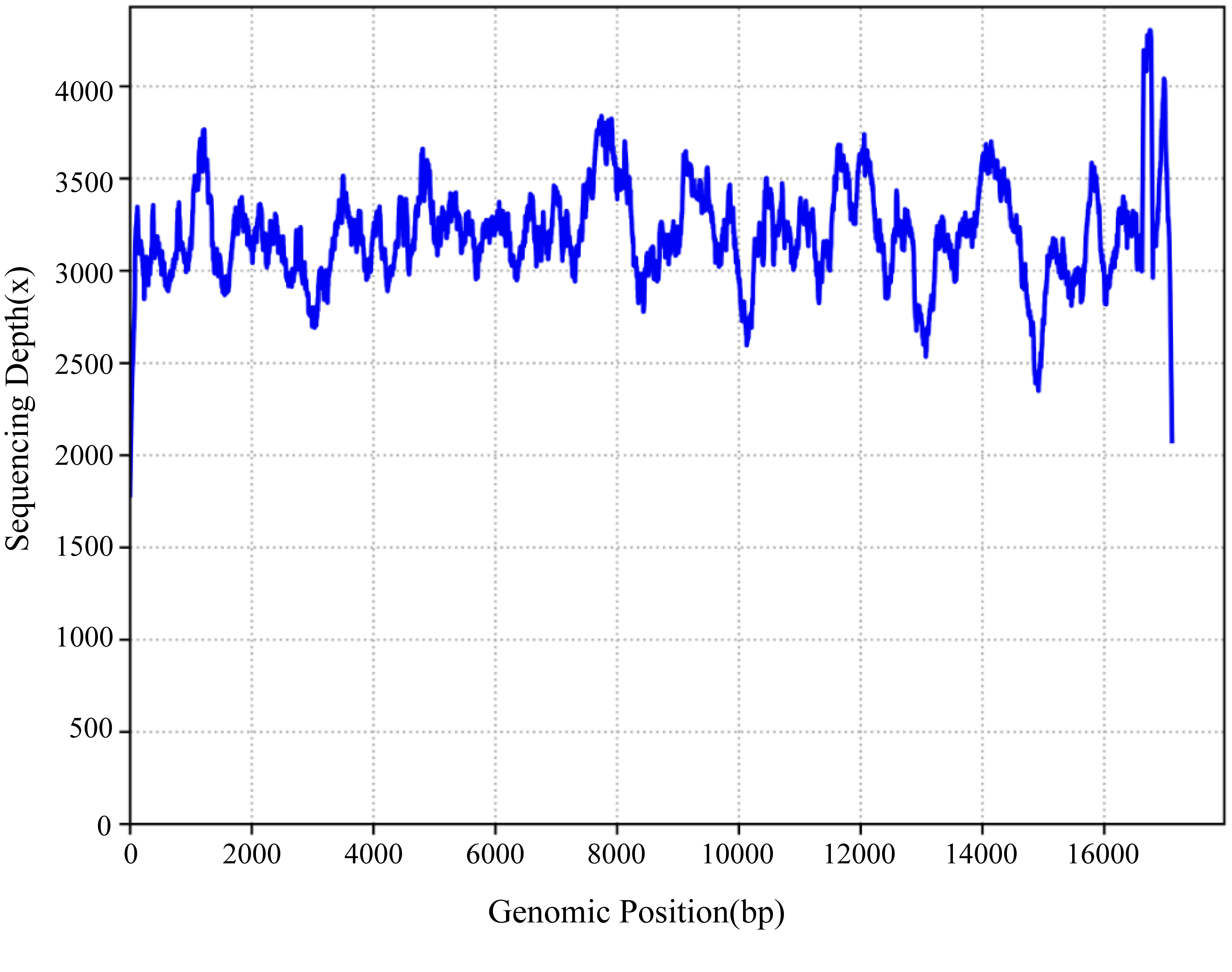

Supplement: Supplementary file 1 — Figure S1: Mitogenome sequencing depth and coverage map of Sorex cansulus. [file ECE3-16-e73766-s011.tif]

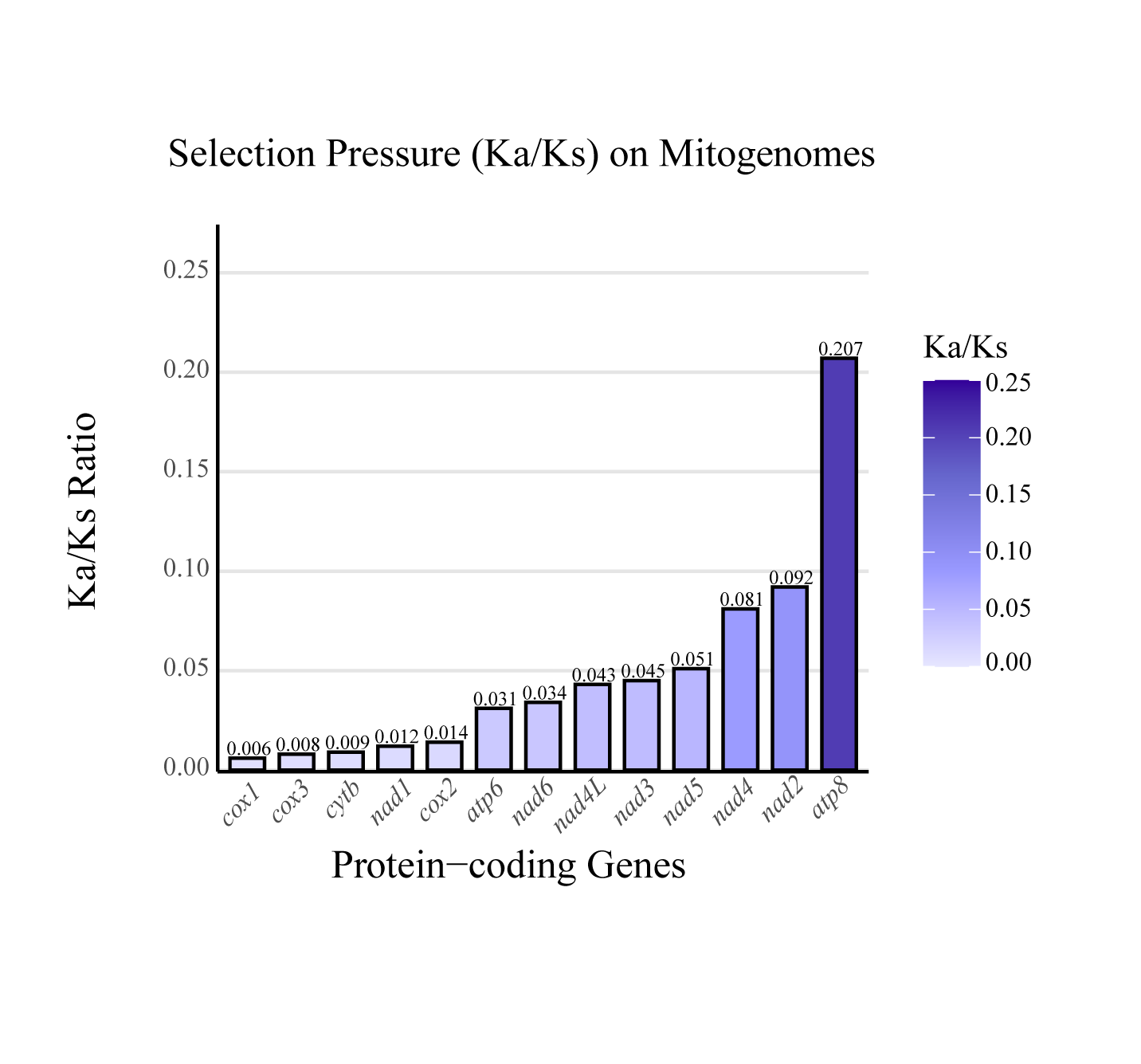

Supplement: Supplementary file 2 — Figure S2: Selection pressure analysis chart of 13 protein‐coding genes of Sorex cansulus. [file ECE3-16-e73766-s010.tif]

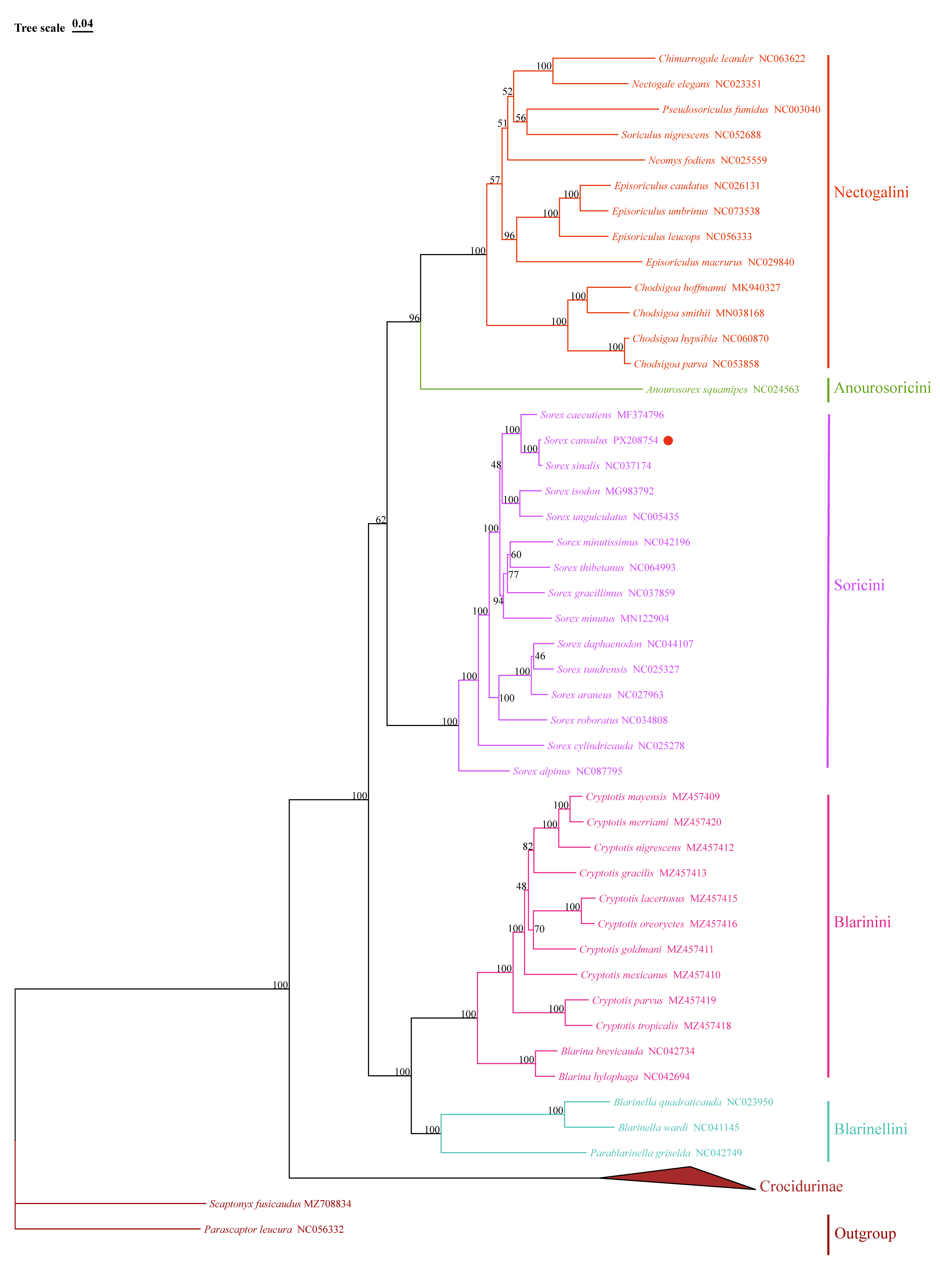

Supplement: Supplementary file 3 — Figure S3: Maximum Likelihood Phylogenetic Tree of the subfamily Soricinae. [file ECE3-16-e73766-s006.tif]
