# Supplementary material for: Insights Into Tribal‐Level Adaptive Evolution and Phylogeny in Soricinae From Mitogenome of the Chinese Endemic Sorex cansulus
Source: Ecol Evol. 2026 Jun 9;16(6):e73766. doi: 10.1002/ece3.73766 (PMC13249582; doi:10.1002/ece3.73766)
Supplement: Supplementary file 4 — Table S1: Species information. [file ECE3-16-e73766-s002.docx]

| Table S1. Species information. | | | | | | |
| --- | --- | --- | --- | --- | --- | --- |
|  | Family | Subfamily | Genus | Species | GenBank  number | Country |
| Outgroup | Talpidae | Talpinae | *Scaptonyx* | *Scaptonyx fusicaudus* | MZ708834 | China |
|  |  |  | *Parascaptor* | *Parascaptor leucura* | NC056332 | China |
| Ingroup | Soricidae | Soricinae | *Sorex* | *Sorex alpinus* | NC087795 | China |
|  |  |  |  | *Sorex cylindricauda* | NC025278 | China |
|  |  |  |  | *Sorex roboratus* | NC034808 | China |
|  |  |  |  | *Sorex araneus* | NC027963 | China |
|  |  |  |  | *Sorex daphaenodon* | NC044107 | China |
|  |  |  |  | *Sorex tundrensis* | NC025327 | China |
|  |  |  |  | *Sorex minutus* | MN122904 | Denmark |
|  |  |  |  | *Sorex gracillimus* | NC037859 | China |
|  |  |  |  | *Sorex minutissimus* | NC042196 | China |
|  |  |  |  | *Sorex thibetanus* | NC064993 | China |
|  |  |  |  | *Sorex isodon* | MG983792 | China |
|  |  |  |  | *Sorex unguiculatus* | NC005435 | Japan |
|  |  |  |  | *Sorex caecutiens* | MF374796 | China |
|  |  |  |  | *Sorex cansulus* | PX208754 | China |
|  |  |  |  | *Sorex sinalis* | NC037174 | China |
|  |  |  | *Neomys* | *Neomys fodiens* | NC025559 | China |
|  |  |  | *Pseudosoriculus* | *Pseudosoriculus fumidus* | NC003040 | China |
|  |  |  | *Chimarrogale* | *Chimarrogale leander* | NC063622 | China |
|  |  |  | *Nectogale* | *Nectogale elegans* | NC023351 | China |
|  |  |  | *Soriculus* | *Soriculus nigrescens* | NC052688 | China |
|  |  |  | *Anourosorex* | *Anourosorex squamipes* | NC024563 | China |
|  |  |  | *Chodsigoa* | *Chodsigoa hoffmanni* | MK940327 | China |
|  |  |  |  | *Chodsigoa smithii* | MN038168 | China |
|  |  |  |  | *Chodsigoa hypsibia* | NC060870 | China |
|  |  |  |  | *Chodsigoa parva* | NC053858 | China |
|  |  |  | *Episoriculus* | *Episoriculus macrurus* | NC029840 | China |
|  |  |  |  | *Episoriculus leucops* | NC056333 | China |
|  |  |  |  | *Episoriculus caudatus* | NC026131 | China |
|  |  |  |  | *Episoriculus umbrinus* | NC073538 | China |
|  |  |  | *Cryptotis* | *Cryptotis parvus* | MZ457419 | USA |
|  |  |  |  | *Cryptotis tropicalis* | MZ457418 | Guatemala |
|  |  |  |  | *Cryptotis mexicanus* | MZ457410 | Mexico |
|  |  |  |  | *Cryptotis goldmani* | MZ457411 | USA |
|  |  |  |  | *Cryptotis lacertosus* | MZ457415 | Guatemala |
|  |  |  |  | *Cryptotis oreoryctes* | MZ457416 | Guatemala |
|  |  |  |  | *Cryptotis gracilis* | MZ457413 | Costa Rica |
|  |  |  |  | *Cryptotis nigrescens* | MZ457412 | Costa Rica |
|  |  |  |  | *Cryptotis mayensis* | MZ457409 | Mexico |
|  |  |  |  | *Cryptotis merriami* | MZ457420 | Guatemala |
|  |  |  | *Parablarine* | *Parablarinella griselda* | NC042749 | China |
|  |  |  | *Blarinella* | *Blarinella quadraticauda* | NC023950 | China |
|  |  |  |  | *Blarinella wardi* | NC041145 | China |
|  |  |  | *Blarina* | *Blarina brevicauda* | NC042734 | Canada |
|  |  |  |  | *Blarina hylophaga* | NC042694 | USA |
|  |  |  |  |  |  |  |
|  |  | Crocidurinae | *Crocidura* | *Crocidura leucodon* | NC070048 | Turkey |
|  |  |  |  | *Crocidura sp s AB 2025* | PV685037 | Ethiopia |
|  |  |  |  | *Crocidura baileyi* | PQ863111 | Ethiopia |
|  |  |  |  | *Crocidura lucina* | PQ863110 | Ethiopia |
|  |  |  |  | *Crocidura glassi* | PQ863109 | Ethiopia |
|  |  |  |  | *Crocidura harenna* | PQ863112 | Ethiopia |
|  |  |  |  | *Crocidura macmillani* | PQ863113 | Ethiopia |
|  |  |  |  | *Crocidura armenica* | OR449074 | Armenia |
|  |  |  |  | *Crocidura shantungensis* | NC021398 | Korea |
|  |  |  |  | *Crocidura sibirica* | MH349094 | China |
|  |  |  |  | *Crocidura suaveolens* | MW815431 | Sevilla |
|  |  |  |  | *Crocidura gueldenstaedtii* | ON682408 | Turkey |
|  |  |  |  | *Crocidura suaveolens mimula* | ON682436 | Turkey |
|  |  |  |  | *Crocidura fuliginosa* | NC042762 | China |
|  |  |  |  | *Crocidura neglecta* | MW815429 | Sevilla |
|  |  |  |  | *Crocidura tanakae* | NC046831 | China |
|  |  |  |  | *Crocidura wuchihensis* | NC079638 | China |
|  |  |  |  | *Crocidura dongyangjiangensis* | NC056167 | China |
|  |  |  |  | *Crocidura lasiura* | NC029329 | Korea |
|  |  |  |  | *Crocidura anhuiensis* | NC088563 | China |
|  |  |  |  | *Crocidura attenuata* | NC026204 | China |
|  |  |  |  | *Crocidura rapax* | NC085826 | China |
|  |  |  |  | *Crocidura nicobarica* | MZ556326 | India |
|  |  |  |  | *Crocidura orientalis* | NC027242 | USA |
|  |  |  |  | *Crocidura malayana* | MW815418 | Sevilla |
|  |  |  |  | *Crocidura palawanensis* | NC027243 | Philippines |
|  |  |  |  | *Crocidura beata* | NC027249 | Philippines |
|  |  |  |  | *Crocidura mindorus* | NC027248 | Philippines |
|  |  |  |  | *Crocidura grayi* | NC027247 | Philippines |
|  |  |  |  | *Crocidura ninoyi* | NC027244 | Philippines |
|  |  |  |  | *Crocidura negrina* | NC027245 | Philippines |
|  |  |  |  | *Crocidura panayensis* | NC027246 | Philippines |
|  |  |  | *Suncus* | *Suncus murinus* | NC024604 | China |
