# Supplementary material for: Insights Into Tribal‐Level Adaptive Evolution and Phylogeny in Soricinae From Mitogenome of the Chinese Endemic Sorex cansulus
Source: Ecol Evol. 2026 Jun 9;16(6):e73766. doi: 10.1002/ece3.73766 (PMC13249582; doi:10.1002/ece3.73766)
Supplement: Supplementary file 5 — Table S2: Fossil calibration points for divergence time trees. [file ECE3-16-e73766-s004.docx]

Table S2. Fossil calibration points for divergence time trees.

| Node | Fossil | Hard lower bound (Ma) | Soft (95%) upper bound (Ma) | Reference |
| --- | --- | --- | --- | --- |
| Soricinae/Crocidurinae | **Eaelist Soricinae and Crocidurinae:**  The oldest Soricinae - Crocidurinae ancestors lived about 20 million years ago (Ma). | 20.0 | 25.0 | (Reumer, 1989 & 1994) |
| Anourosoricini/Nectogalini | **Earliest Anourosoricini:**  *Crusafontina exculta* and *Darocasorex vandermeuleni* - early Late Miocene localities of Central Europe (11.5–11 Ma); *Crusafontina* aff. *endemica* and *Crusafontina endemica* from Rudábanya MN9 (ca. 10 Ma) | 11.0 | 20.0 | (Prieto & Van Dam, 2016) |
| Blarinini / Blarinellini | **Earliest Blarinini and Blarinellini:**  The oldest Blarinellini - Europe in the Early Miocene, Hemisorex sp. (MN3-4)  The oldest Blarinini - Middle-Late Miocene of NA  The oldest Blarinini: 16.3 -13.6 Ma | 12.6 | 20.0 | (Rzebik-Kowalska, 1998)  (Harris, 1998)  (Repenning, 1967)  (Gunnel, & Bloch, 2007) |
| Separation of *Sorex minutus* group from other *Sorex* | **Earliest fossil attributed to *Sorex minutus*:**  The divergence of this lineage (*Sorex minutoides* - *Sorex minutus*) from other *Sorex* took place in Late Miocene | 4.2 | 8.7 | (Storch et al., 1998)  (Rzebik-Kowalska, 1998; 2007) |
| *Sorex daphaenodon / Sorex tundrensis* | ***Sorex daphaenodon* in Yakutia:**  The fossils of *Sorex daphaenodon* 0.8–0.95 Ma | 0.8 | 1.8 | (Sher 1971; 1984; Virina *et al* 1984) |
