# Supplementary material for: Insights Into Tribal‐Level Adaptive Evolution and Phylogeny in Soricinae From Mitogenome of the Chinese Endemic Sorex cansulus
Source: Ecol Evol. 2026 Jun 9;16(6):e73766. doi: 10.1002/ece3.73766 (PMC13249582; doi:10.1002/ece3.73766)
Supplement: Supplementary file 6 — Table S3: Nucleotide composition of the Sorex cansulus mitogenome. [file ECE3-16-e73766-s001.docx]

Table S3. Nucleotide composition of the *Sorex cansulus* mitogenome.

| Regions | Size (bp) | T(U)% | C% | A% | G% | AT (%) | GC (%) | AT skew | GC skew |
| --- | --- | --- | --- | --- | --- | --- | --- | --- | --- |
| Mitogenome | 17115 | 29.4 | 24.5 | 32.9 | 13.2 | 62.3 | 37.7 | 0.057 | -0.301 |
| PCGs | 11412 | 30.5 | 25.5 | 31 | 13 | 61.5 | 38.5 | 0.007 | -0.324 |
| rRNAs | 2536 | 25.7 | 20.8 | 36.2 | 17.3 | 61.9 | 38.1 | 0.169 | -0.093 |
| tRNAs | 1516 | 31.9 | 16 | 33.4 | 18.6 | 65.3 | 34.6 | 0.023 | 0.074 |
| OL | 33 | 15.2 | 24.2 | 33.3 | 27.3 | 48.5 | 51.5 | 0.375 | 0.059 |
| D-loop | 1659 | 32.4 | 22.2 | 33.2 | 12.1 | 65.6 | 34.4 | 0.012 | -0.295 |
