# Supplementary material for: Insights Into Tribal‐Level Adaptive Evolution and Phylogeny in Soricinae From Mitogenome of the Chinese Endemic Sorex cansulus
Source: Ecol Evol. 2026 Jun 9;16(6):e73766. doi: 10.1002/ece3.73766 (PMC13249582; doi:10.1002/ece3.73766)
Supplement: Supplementary file 7 — Table S4: Codon frequency and relative rynonymous codon usage (RSCU) of protein‐coding genes (PCGs) in the Sorex cansulus mitogenome. [file ECE3-16-e73766-s005.docx]

Table S4. Codon frequency and relative rynonymous codon usage (RSCU) of protein-coding genes (PCGs) in the *Sorex cansulus* mitogenome.

| **Codon** | **Count** | **RSCU** | **Codon** | **Count** | **RSCU** | **Codon** | **Count** | **RSCU** | **Codon** | **Count** | **RSCU** |
| --- | --- | --- | --- | --- | --- | --- | --- | --- | --- | --- | --- |
| **GCU** | **62** | **1** | **GGG** | **19** | **0.35** | **AAU** | **63** | **0.77** | **UCG** | **5** | **0.1** |
| **GCC** | **69** | **1.12** | **CAU** | **28** | **0.61** | **AAC** | **101** | **1.23** | **AGU** | **18** | **0.37** |
| **GCA** | **112** | **1.81** | **CAC** | **64** | **1.39** | **CCU** | **67** | **1.43** | **AGC** | **34** | **0.71** |
| **GCG** | **4** | **0.06** | **AUU** | **198** | **1.14** | **CCC** | **39** | **0.83** | **ACU** | **62** | **0.83** |
| **UGU** | **10** | **0.8** | **AUC** | **148** | **0.86** | **CCA** | **78** | **1.67** | **ACC** | **80** | **1.07** |
| **UGC** | **15** | **1.2** | **AAA** | **88** | **1.83** | **CCG** | **3** | **0.06** | **ACA** | **145** | **1.95** |
| **GAU** | **28** | **0.81** | **AAG** | **8** | **0.17** | **CAA** | **78** | **1.73** | **ACG** | **11** | **0.15** |
| **GAC** | **41** | **1.19** | **UUA** | **132** | **1.3** | **CAG** | **12** | **0.27** | **GUU** | **57** | **1.16** |
| **GAA** | **82** | **1.67** | **UUG** | **18** | **0.18** | **CGU** | **9** | **0.56** | **GUC** | **35** | **0.71** |
| **GAG** | **16** | **0.33** | **CUU** | **91** | **0.9** | **CGC** | **8** | **0.5** | **GUA** | **94** | **1.92** |
| **UUU** | **101** | **0.87** | **CUC** | **84** | **0.83** | **CGA** | **44** | **2.75** | **GUG** | **10** | **0.2** |
| **UUC** | **132** | **1.13** | **CUA** | **255** | **2.52** | **CGG** | **3** | **0.19** | **UGA** | **98** | **1.88** |
| **GGU** | **48** | **0.89** | **CUG** | **27** | **0.27** | **UCU** | **52** | **1.08** | **UGG** | **6** | **0.12** |
| **GGC** | **58** | **1.07** | **AUA** | **175** | **1.54** | **UCC** | **73** | **1.52** | **UAU** | **78** | **1.07** |
| **GGA** | **91** | **1.69** | **AUG** | **53** | **0.46** | **UCA** | **107** | **2.22** | **UAC** | **68** | **0.93** |

Notes: Termination codons were not included in the analysis. Overrepresented codons (RSCU > 1.6) are highlighted in red, and underrepresented codons (RSCU < 0.6) are showed in blue.
