# Supplementary material for: Insights Into Tribal‐Level Adaptive Evolution and Phylogeny in Soricinae From Mitogenome of the Chinese Endemic Sorex cansulus
Source: Ecol Evol. 2026 Jun 9;16(6):e73766. doi: 10.1002/ece3.73766 (PMC13249582; doi:10.1002/ece3.73766)
Supplement: Supplementary file 8 — Table S5: Ka/Ks ratios of protein‐coding genes (PCGs) in 5 tribes of the subfamily Soricinae. [file ECE3-16-e73766-s012.docx]

Table S5. Ka/Ks ratios of protein-coding genes (PCGs) in 5 tribes of the subfamily Soricinae.

| Tribes | Ka/Ks | | | | | | | | | | | | | |
| --- | --- | --- | --- | --- | --- | --- | --- | --- | --- | --- | --- | --- | --- | --- |
|  | *atp8* | *atp6* | *cox1* | *cox2* | *cox3* | *cytb* | *nad1* | *nad2* | *nad3* | *nad4* | *nad4L* | *nad5* | *nad6* | mean |
| Nectogalini | 0.212 | 0.018 | 0.006 | 0.013 | 0.007 | 0.009 | 0.014 | 0.094 | 0.055 | 0.046 | 0.042 | 0.049 | 0.048 | 0.047 |
| Anourosoricini | 0.187 | 0.023 | 0.006 | 0.015 | 0.011 | 0.025 | 0.014 | 0.051 | 0.078 | 0.029 | 0.048 | 0.082 | 0.053 | 0.048 |
| Soricini | 0.150 | 0.022 | 0.006 | 0.014 | 0.009 | 0.009 | 0.016 | 0.106 | 0.046 | 0.069 | 0.052 | 0.059 | 0.036 | 0.046 |
| Blarrinini | 0.086 | 0.021 | 0.006 | 0.015 | 0.018 | 0.013 | 0.026 | 0.088 | 0.059 | 0.043 | 0.041 | 0.059 | 0.037 | 0.039 |
| Blarinellini | 0.173 | 0.015 | 0.006 | 0.014 | 0.013 | 0.018 | 0.020 | 0.060 | 0.035 | 0.048 | 0.038 | 0.047 | 0.052 | 0.041 |
| mean | 0.162 | 0.020 | 0.006 | 0.014 | 0.012 | 0.015 | 0.018 | 0.080 | 0.055 | 0.047 | 0.044 | 0.059 | 0.045 |  |
