# Supplementary material for: Insights Into Tribal‐Level Adaptive Evolution and Phylogeny in Soricinae From Mitogenome of the Chinese Endemic Sorex cansulus
Source: Ecol Evol. 2026 Jun 9;16(6):e73766. doi: 10.1002/ece3.73766 (PMC13249582; doi:10.1002/ece3.73766)
Supplement: Supplementary file 9 — Table S6: Saturation test of nucleotide sequence for protein‐coding genes (PCGs) of Sorex cansulus . [file ECE3-16-e73766-s008.docx]

Table S6. Saturation test of nucleotide sequence for protein-coding genes (PCGs) of *Sorex cansulus*.

| NumOTU | Iss | Iss.cSym | T | DF | P | Iss.cAym | T | DF | P |
| --- | --- | --- | --- | --- | --- | --- | --- | --- | --- |
| 4 | 0.518 | 0.850 | 41.478 | 4532 | 0.000 | 0.841 | 40.343 | 4532 | 0.000 |
| 8 | 0.519 | 0.845 | 39.484 | 4532 | 0.000 | 0.765 | 29.783 | 4532 | 0.000 |
| 16 | 0.518 | 0.830 | 37.755 | 4532 | 0.000 | 0.677 | 19.243 | 4532 | 0.000 |
| 32 | 0.520 | 0.810 | 35.222 | 4532 | 0.000 | 0.562 | 5.159 | 4532 | 0.000 |
