# Supplementary material for: Insights Into Tribal‐Level Adaptive Evolution and Phylogeny in Soricinae From Mitogenome of the Chinese Endemic Sorex cansulus
Source: Ecol Evol. 2026 Jun 9;16(6):e73766. doi: 10.1002/ece3.73766 (PMC13249582; doi:10.1002/ece3.73766)
Supplement: Supplementary file 10 — Table S7: Ka/Ks comparison of protein‐coding genes (PCGs) among Sorex cansulus , Dacnomys millardi , and Soricinae. [file ECE3-16-e73766-s007.docx]

Table S7. Ka/Ks comparison of protein-coding genes (PCGs) among *Sorex cansulus*, *Dacnomys millardi*, and Soricinae.

| **Ka/ks** | | | |  |
| --- | --- | --- | --- | --- |
| **Gene** | ***Sorex cansulus*** | **Soricinae (mean)** | ***Dacnomys millardi*** |  |
| ***cox1*** | 0.006 | 0.009 | 0.011 |  |
| ***cox3*** | 0.008 | 0.013 | 0.051 |  |
| ***cytb*** | 0.009 | 0.014 | 0.065 |  |
| ***nad1*** | 0.012 | 0.020 | 0.088 |  |
| ***cox2*** | 0.014 | 0.015 | 0.029 |  |
| ***atp6*** | 0.031 | 0.024 | 0.064 |  |
| ***nad6*** | 0.034 | 0.040 | 0.230 |  |
| ***nad4L*** | 0.043 | 0.060 | 0.117 |  |
| ***nad3*** | 0.045 | 0.053 | 0.063 |  |
| ***nad5*** | 0.051 | 0.062 | 0.156 |  |
| ***nad4*** | 0.081 | 0.052 | 0.120 |  |
| ***nad2*** | 0.092 | 0.088 | 0.273 |  |
| ***atp8*** | 0.207 | 0.153 | 0.570 |  |
| **mean** | 0.049 | 0.046 | 0.141 |  |
