# Supplementary material for: Insights Into Tribal‐Level Adaptive Evolution and Phylogeny in Soricinae From Mitogenome of the Chinese Endemic Sorex cansulus
Source: Ecol Evol. 2026 Jun 9;16(6):e73766. doi: 10.1002/ece3.73766 (PMC13249582; doi:10.1002/ece3.73766)
Supplement: Supplementary file 11 — Table S8: Genetic distance between Pseudosoriculus and Episoriculus. [file ECE3-16-e73766-s003.docx]

Table S8. Genetic distance between *Pseudosoriculus* and *Episoriculus.*

|  | *Pseudosoriculus fumidus* | *Episoriculus caudatus* | *Episoriculus umbrinus* | *Episoriculus leucops* | *Episoriculus macrurus* |
| --- | --- | --- | --- | --- | --- |
| *Pseudosoriculus fumidus* |  |  |  |  |  |
| *Episoriculus caudatus* | 0.1959 |  |  |  |  |
| *Episoriculus umbrinus* | 0.1878 | 0.0834 |  |  |  |
| *Episoriculus leucops* | 0.2005 | 0.1362 | 0.1236 |  |  |
| *Episoriculus macrurus* | 0.1800 | 0.1813 | 0.1717 | 0.1732 |  |
