# Supplementary material for: Insights Into Tribal‐Level Adaptive Evolution and Phylogeny in Soricinae From Mitogenome of the Chinese Endemic Sorex cansulus
Source: Ecol Evol. 2026 Jun 9;16(6):e73766. doi: 10.1002/ece3.73766 (PMC13249582; doi:10.1002/ece3.73766)
Supplement: Supplementary file 12 — Table S9: Genetic distance between Parablarinella and Blarinella. [file ECE3-16-e73766-s009.docx]

Table S9. Genetic distance between *Parablarinella* and *Blarinella.*

|  | *Parablarinella griselda* | *Blarinella quadraticauda* | *Blarinella wardi* |
| --- | --- | --- | --- |
| *Parablarinella griselda* |  |  |  |
| *Blarinella quadraticauda* | 0.201 |  |  |
| *Blarinella wardi* | 0.204 | 0.132 |  |
